# Bacterial Evolutionary Precursors of Eukaryotic Copper–Zinc Superoxide Dismutases

**DOI:** 10.1093/molbev/msab157

**Published:** 2021-05-22

**Authors:** Gareth S A Wright

**Affiliations:** Department of Biochemistry & Systems Biology, Institute of Systems, Molecular and Integrative Biology, University of Liverpool, Liverpool, United Kingdom

**Keywords:** metallochaperone, eukaryogenesis, oxidative stress, protein interface, SOD1

## Abstract

Internalization of a bacteria by an archaeal cell expedited eukaryotic evolution. An important feature of the species that diversified into the great variety of eukaryotic life visible today was the ability to combat oxidative stress with a copper–zinc superoxide dismutase (CuZnSOD) enzyme activated by a specific, high-affinity copper chaperone. Adoption of a single protein interface that facilitates homodimerization and heterodimerization was essential; however, its evolution has been difficult to rationalize given the structural differences between bacterial and eukaryotic enzymes. In contrast, no consistent strategy for the maturation of periplasmic bacterial CuZnSODs has emerged. Here, 34 CuZnSODs are described that closely resemble the eukaryotic form but originate predominantly from aquatic bacteria. Crystal structures of a *Bacteroidetes bacterium* CuZnSOD portray both prokaryotic and eukaryotic characteristics and propose a mechanism for self-catalyzed disulfide maturation. Unification of a bacterial but eukaryotic-like CuZnSOD along with a ferredoxin-fold MXCXXC copper-binding domain within a single polypeptide created the advanced copper delivery system for CuZnSODs exemplified by the human copper chaperone for superoxide dismutase-1. The development of this system facilitated evolution of large and compartmentalized cells following endosymbiotic eukaryogenesis.

## Introduction

Superoxide dismutases and reductases are deployed almost ubiquitously throughout nature to prevent damage to biological molecules by superoxide (Craig and Slauch 2009; Gu and Imlay 2013; Lu et al. 2018). They employ redox-active metal cofactors to catalyze the production of molecular oxygen and hydrogen peroxide from superoxide. Iron, manganese, nickel, and copper can all serve this purpose. Iron and manganese superoxide dismutase isoenzymes evolved in bacteria prior to oxidation of the Earth’s atmosphere (Dupont et al. 2010; Inupakutika et al. 2016). CuZn superoxide dismutases (CuZnSODs) evolved independently following atmospheric and oceanic oxidation as copper and zinc became bioavailable (Zerkle et al. 2005; Harel et al. 2014).

CuZnSODs from *Photobacterium leiognathi* (*Ph*CuZnSOD) (Bourne et al. 1996), *Salmonella enterica serovar typhimurium* (Pesce et al. 2000), *Mycobacterium tuberculosiss* (Spagnolo et al. 2004), and *Actinobacillus pleuropneumoniae* (Forest et al. 2000) form P-class homodimers through interface–cavity water network interactions. The P-class interface is not conserved across prokaryotic CuZnSODs, however, with P-class-like but monomeric examples found in *Escherichia coli* (Battistoni et al. 1996), *Salmonella enterica serovar choleraesuis* (Mori et al. 2008), and *Brucella abortus* (Pratt et al. 2015). These periplasmic bacterial CuZnSODs account for a small fraction of cellular superoxide dismutase activity (Steinman 1987; Benov and Fridovich 1994). In contrast, CuZnSODs are the most abundant eukaryotic superoxide detoxifier and operate within the cytoplasm, peroxisomes, mitochondrial intermembrane space, extracellular space, and nucleus (Marklund 1982; Crapo et al. 1992; Sturtz et al. 2001). They are found in animals (McCord and Fridovich 1969), plants (Sawada et al. 1972), fungi (Goscin and Fridovich 1972), and protists (Ferro et al. 2015). Each conforms to a conserved, hydrophobic E-class dimer interface that incorporates four strong intersubunit hydrogen bonds (Richardson et al. 1975; Djinović et al. 1991; Lartigue et al. 2015). E-class and P-class dimerization are mechanistically distinct and the interfaces are found on opposite poles of the CuZnSOD β-barrel (Bourne et al. 1996).

Following translation, a sequence of post-translational modifications transform disordered, monomeric polypeptide into active CuZnSOD homodimers. Folding, zinc and copper binding, and acquisition of an intrasubunit disulfide bond can all be assisted by chaperones (Culotta et al. 1997; Battistoni et al. 1999; Osman et al. 2013; Luchinat et al. 2017; Wright 2020). For example, approximately 80% of human CuZn-superoxide dismutase-1 (SOD1) is activated by the human copper chaperone for SOD1 (hCCS) (Subramaniam et al. 2002). In addition, CCS orchestrates CuZnSOD localization to the mitochondrial intermembrane space and peroxisomes (Field et al. 2003; Kawamata and Manfredi 2008; Islinger et al. 2009). hCCS functionality rests on its ability to form E-class heterodimers with SOD1 (Schmidt et al. 1999; Banci et al. 2012). This is mediated by an hCCS domain with 50% identity to SOD1 that conserves protein fold, zinc binding and intrasubunit disulfide bonding (Lamb et al. 2000; Sala et al. 2019). To facilitate metalation of its CuZnSOD substrate, hCCS also contains a ferredoxin-fold, MXCXXC copper-binding domain similar in structure and function to eukaryotic Atx1, prokaryotic CopZ, and related domains within the copper-transporting ATPases. Therefore, eukaryotic CuZnSOD copper chaperones likely arose through gene duplication followed by domain shuffling and sequence optimization. While intracellular E-class CuZnSOD-cognate copper chaperone pairs are very well conserved across eukaryotes they are completely absent from prokaryotes including Asgard archaea and, specifically, the Lokiarchaeota species closely related to eukaryotes (Spang et al. 2015). Thus, eukaryotic CuZnSODs and their cognate chaperone differentiated after adoption of the E-class interface but before eukaryotic radiation. Evolution of eukaryotic CuZnSODs and their chaperones has however been difficult to reconcile due to the deep division in the sequence, structure, and activation mechanisms of bacterial and eukaryotic CuZnSODs characterized thus far.

Bridging the divide between eukaryotic and prokaryotic enzymes, here I describe several classes of bacterial CuZnSODs that are more similar to the eukaryotic form than they are to previously characterized bacterial enzymes. The CuZnSOD of a *Bacteroidetes bacterium* forms its intrasubunit disulfide bond quickly and independently of chaperones. Crystal structures of this distinctly E-class protein show it includes P-class structural elements positioning several of these bacterial CuZnSODs as an evolutionary stepping-stone between E-class and P-class enzymes. The E-class interface is therefore of bacterial origin and this group of bacterial CuZnSODs are precursors of eukaryotic CuZnSOD-cognate copper chaperone pairs necessary for the development of complex eukaryotic cellular structure following endosymbiotic eukaryogenesis (Fan et al. 2020).

## Results

### Discovery of a New Group of Bacterial CuZnSODs

**Fig. 1.**
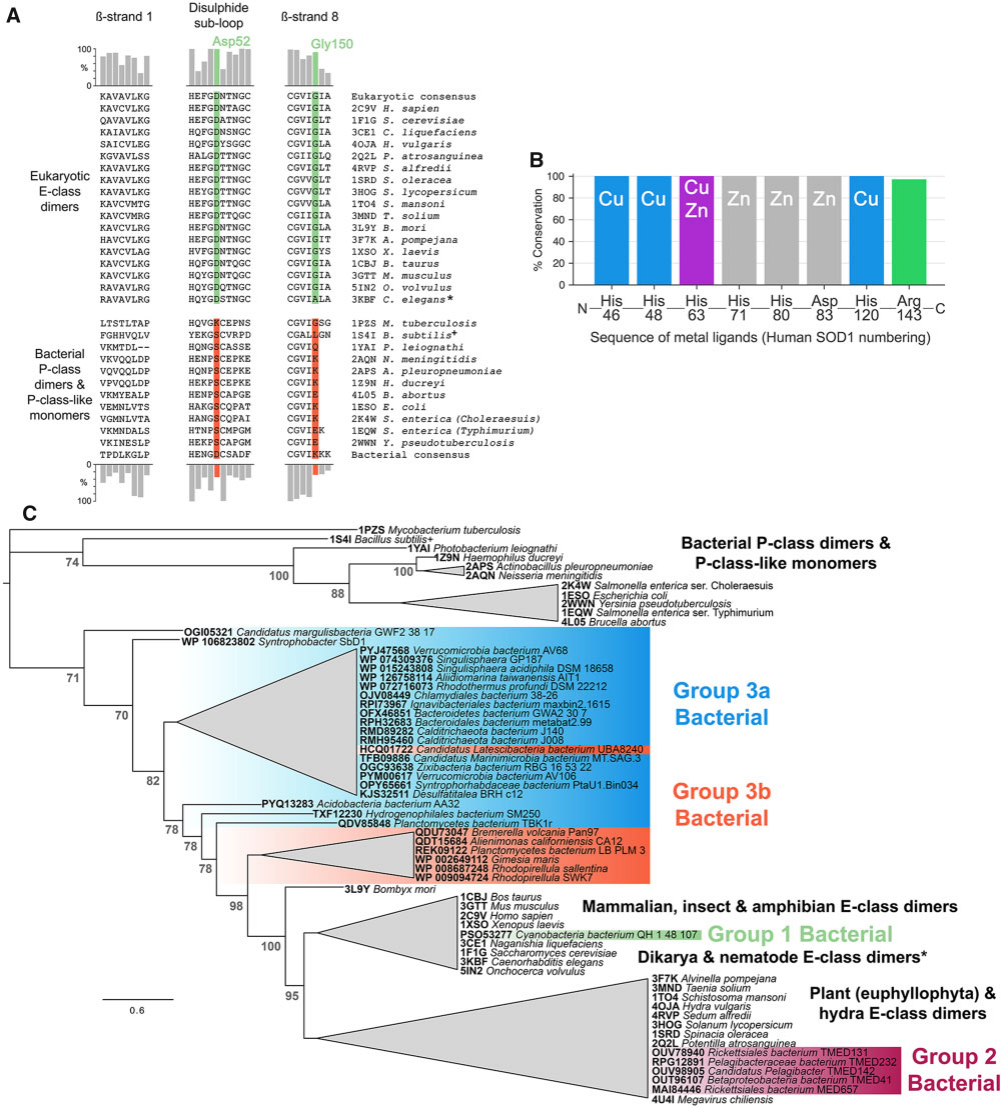
Eukaryotic-like CuZnSODs are present in bacteria and can be separated into three groups. (*A*) Comparison of homodimer interface amino acids found in those bacterial and eukaryotic CuZnSODs represented by structures present in the PDB. Bar charts indicate amino acid conservation with respect to eukaryotic (upper) and bacterial (lower) consensus sequences constructed from 378 eukaryotic and 248 bacterial sequences retrieved from the OMA database (Altenhoff et al. 2018). (*B*) Bacterial E-class-like CuZnSODs have conserved metal-binding ligand type and order in comparison with other eukaryotic and bacterial enzymes. Catalytically important Arg143 (human numbering) is also very highly conserved with one exception: *Candidatus Margulisbacteria bacterium* GWF2_38_17 CuZnSOD OGI05321 has proline substitution at this site. (*C*) Unrooted phylogenetic tree showing eukaryotic-like bacterial CuZnSODs clustered in three groups within the E-class clade rather than with bacterial P-class dimer and P-class-like monomers. Node numbers in gray represent Bayesian posterior probabilities as percentages. Generated using the Jones–Taylor–Thornton substitution model (supplementary fig. S1*A*, Supplementary Material online). Branches are collapsed where clade topology is unstable across phylogenetic trees generated using different amino acid substitution models (supplementary fig. S1, Supplementary Material online) or where support values are less the 70%. Branch lengths represent expected substitutions per site with a scale bar at the bottom left. * *C. elegans* CuZnSOD (3KBF) has Ala153 in place of human SOD1 Gly150 and as a result the enzyme is monomeric. ^+^ Structure 1S4I from *B. subtilis* is not an active superoxide dismutase as it cannot bind copper but retains a CuZnSOD-like structure.

Bacterial and eukaryotic CuZnSOD structures available in the Protein Data Bank (PDB) have dissimilar β-strands 1 and 8; disulfide subloops particularly in the interface region between Gly51 and Cys57; and do not maintain a glycine residue at the human SOD1 Gly150 position (fig. 1*A*). In the absence of E-class dimerization, the bacterial enzymes have not been subjected to a selective pressure to adopt or maintain E-class interface residues. However, 34% of bacterial CuZnSOD sequences present in the OMA database (Altenhoff et al. 2018) retain an aspartate at the human Asp52 position within the Gly-Asp-X-Thr/Ser disulfide subloop tetrad known to report on eukaryotic CuZnSOD E-class homodimerization (fig. 1*A*) (Culik et al. 2018). This indicates our understanding of bacterial CuZnSOD structure may not be complete.

A protein BLAST search restricted to prokaryotic sequences using the eukaryotic CuZnSOD consensus sequence as bait yielded 34 eukaryotic-like bacterial CuZnSODs found only in gram-negative species and distributed across 12 phyla (supplementary tables S1–S5, Supplementary Material online). Iron and manganese SOD coding sequences are often present in the same genome as P-class CuZnSODs (Benov and Fridovich 1994; Sadosky et al. 1994) and this is also observed for bacterial but eukaryotic-like CuZnSODs (Supplementary tables S1–S4, Supplementary Material online). The majority of these sequences were retrieved by environmental sampling of aquatic environments, seas, and oceans, but also water-permeating rock formations and soil. Several examples have been sequenced following laboratory culture including an example recovered from a marine hydrothermal vent (Rensink et al. 2020). Geographical sampling data indicate eukaryotic-like bacterial CuZnSODs are present globally.

**Fig. 2.**
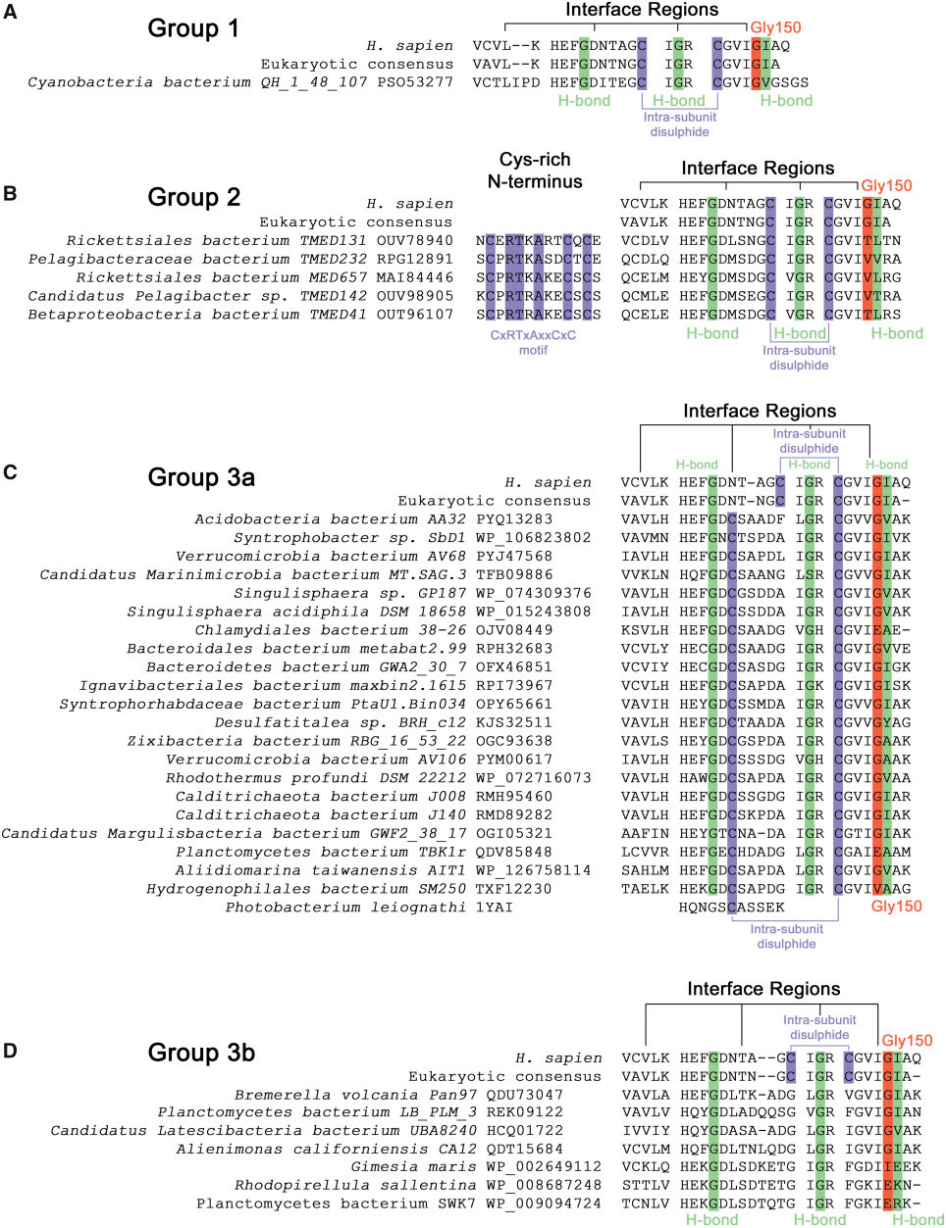
Three groups of eukaryotic-like bacterial CuZnSODs have distinct primary structure features. (*A*) Group 1 *Cyanobacteria bacterium* QH_1_48_107 *Cb*CuZnSOD has E-class intrasubunit disulfide configuration and interface regions with conservative nonpolar substitutions in interface and intersubunit hydrogen bonding residues. (*B*) Group 2 enzymes have an N-terminal CxRTxAxxCxC motif, E-class cysteine configuration, conserved hydrogen bonding residues but large and polar substitutions in place of human Gly150 and β-strand 1, respectively. (*C*) Group 3a interface residues including Gly150 (81% conserved) and intrasubunit hydrogen bonding residues are well conserved from eukaryotic CuZnSODs. Disulfide subloop cysteines involved in intrasubunit disulfide formation are conserved with respect to P-class *P. leiognathi* CuZnSOD (Bourne et al. 1996). (*D*) Group 3b enzymes do not have intrasubunit disulfide bonding cysteines. *G. maris*, *R. sallentina*, and *P. bacterium* SWK7 have poorly conserved E-class interface residues in contrast to other members. All include extended N-termini even after removal of signal peptide but do not contain a CXC motif unlike Group 2 enzymes. Green—interface hydrogen bonding residues, orange—Gly150 equivalent residues (human numbering) (Sala et al. 2019), purple—cysteine.

The 34 bacterial CuZnSODs described in supplementary tables S1–S4, Supplementary Material online have metal-binding residues conserved in both amino acid type and order within the primary sequence compared with other eukaryotic and prokaryotic CuZnSODs confirming a monophyletic origin (fig. 1*B*) (Forest et al. 2000). Overall phylogenetic tree topology was stable across different amino acid substitution models, tree construction methodologies and degree of multiple sequence alignment trimming (supplementary figs. S1–S6, Supplementary Material online) with the exceptions of *Bacillus subtilis* (supplementary figs. S2, S3, and S6, Supplementary Material online) and *Megavirus chiliensis* CuZnSODs positioning (supplementary figs. S1, S3, and S4, Supplementary Material online) which have mutations that prevent copper and zinc binding, respectively. Each parameter set resulted in consensus trees with eukaryotic-like bacterial CuZnSOD groups nested within the eukaryotic CuZnSOD clade (fig. 1*C* and supplementary figs. S1–S6, Supplementary Material online). Indeed, figure 1*C* indicates bacterial eukaryotic-like CuZnSODs share a common ancestor with the eukaryotic E-class enzymes. As a whole, these CuZnSODs do not display P-class interfaces as exemplified by *Ph*CuZnSOD (supplementary fig. S7, Supplementary Material online). However, conservation of eukaryotic interface surfaces, hydrogen bonding residues, and Gly150 (human numbering) indicates several of these bacterial enzymes form E-class homodimers (fig. 2). Eukaryotic-like bacterial CuZnSODs form three groups within the eukaryotic clade, each with distinct features (supplementary table S6, Supplementary Material online).

### Multiple Subgroups of Eukaryotic-Like Bacterial CuZnSODs

Group 1 comprises a single protein (supplementary table S1, Supplementary Material online) encoded within the genome of *Cyanobacterium bacterium* QH_1_48_107 (PSO53277, *Cb*CuZnSOD) discovered at multiple locations in the Chilean Andes (Finstad et al. 2017). No nonbacterial protein coding sequences could be found in the parent whole-genome shotgun (WGS) sequence data (supplementary dataset 1, Supplementary Material online) including coding sequences immediately adjacent to the *Cb*CuZnSOD gene (supplementary table S7, Supplementary Material online). Coding sequence GC content is also well within the genomic DNA normal distribution (supplementary fig. S8, Supplementary Material online). *Cb*CuZnSOD has high amino acid identity to the eukaryotic CuZnSODs consensus sequence (65.4%), but low identity to *Ph*CuZnSOD (30.8%), and similar polypeptide length and predicted mass compared with intracellular, eukaryotic CuZnSODs including human SOD1 (supplementary table S1, Supplementary Material online). Like Group 2 enzymes below, its position within the eukaryotic clade (fig. 1*C*) indicates this bacterial CuZnSOD may result from a lateral gene transfer event from a eukaryotic source. It has E-class dimer interfaces with only conservative nonpolar for nonpolar substitutions found in β-strands 1 and 8 (fig. 2*A*). It very likely maintains the four intersubunit hydrogen bonds found in eukaryotic CuZnSODs (fig. 2*A*). The position of the intrasubunit disulfide bonding cysteines indicates a eukaryotic configuration linking β-strand 8 to the upper disulfide subloop (fig. 2*A*). Absence of any signal peptide to facilitate transportation through the periplasmic membrane also indicates *Cb*CuZnSOD is located to the cytoplasm unlike all bacterial CuZnSODs described to date.

Group 2 *Proteobacterial* enzymes are 54.5% ± 2.3 identical to the eukaryotic consensus CuZnSOD and 32.8% ± 1.9 identical to *Ph*CuZnSOD (supplementary table S2, Supplementary Material online). *Rickettsiales bacterium* TMED131 sequence data, which encodes *Rb*CuZnSOD (OUV78940), was assessed for contamination by non-bacterial sources. Despite extensive bacteriophage transduction no eukaryotic or eukaryotic virus contamination was found (supplementary dataset 2 and table S8, Supplementary Material online). Group 2 CuZnSODs differ from the eukaryotic form and *Cb*CuZnSOD, in that they have extended, cysteine-rich, N-termini CxRTxAxxCxC motifs containing the unusual CXC triad found in the C-terminal domain of many eukaryotic CCS proteins (fig. 2*B*). The CXC motif binds copper (Allen et al. 2012) indicating a possible enzymatic activation function similar to the N-terminal histidine rich extensions found in some P-class CuZnSODs (Battistoni et al. 2001) or a role in disulfide formation (Banci et al. 2012). While this group largely maintains the residues involved in intersubunit hydrogen bonding, the N-terminal portion of the E-class interface is poorly conserved (fig. 2*B*). In addition, all of these enzymes have threonine or valine substitutions at sites analogous to human SOD1 Gly150 (fig. 2*B*) where even small side-chain substitutions reduce E-class homodimer affinity (Sala et al. 2019). These enzymes are therefore eukaryotic-like but predicted to be monomeric.

Group 3a is the most populous with 21 members. Mean identity to the eukaryotic consensus is 51.3% ± 3.1 whereas identity to *Ph*CuZnSOD is 33.3% ± 2.5 (supplementary table S3, Supplementary Material online). *Bacteroidetes bacterium* GWA2_30_7 (*Bb*CuZnSOD) was assessed for WGS sequence contamination from nonbacterial sources (supplementary dataset 3 and table S9, Supplementary Material online). Like P-class bacterial CuZnSODs, Group 3a leader sequences indicate they are found within the periplasmic space with one example anchored via a trans-membrane helix. Group 3a CuZnSODs as a whole have conserved E-class N- and C-terminal interface regions but their disulfide subloop has an unusual Gly-Asp-Cys-Thr/Ser interface tetrad (fig. 2*C*). This cysteine positioning is ubiquitously found in P-class and P-class-like bacterial CuZnSODs but never in E-class enzymes.

Group 3b enzymes are a heterogenous group that belong, in the main, to the *Planctomycetes* phylum of budding bacteria (supplementary table S4, Supplementary Material online). Phylogenetic analysis indicates Group 3b is closely related to Group 3a (fig. 1*C*). They have mean 54.5% ± 2.7 identity to the eukaryotic CuZnSOD consensus and 35.2% ± 1.6 identity to *Ph*CuZnSOD. Five are predicted to be secreted to the periplasm while two are cytoplasmic (supplementary table S4, Supplementary Material online). Their unifying characteristic is the lack of intrasubunit disulfide bonding cysteines (fig. 2*D*). The CuZnSOD disulfide is very well conserved across CuZnSODs with the exception of some protist enzymes (Ferro et al. 2015) but here it is replaced with a hydrophobic interaction also seen in the SOD1-like domain of *Saccharomyces cerevisiae* yCCS (fig. 2*D*) (Lamb et al. 1999). Four-of-seven enzymes maintain a glycine residue at the human Gly150 position, E-class interface regions and intersubunit hydrogen bonding residues. Despite this, dimer affinity is very likely to be reduced by the inability to covalently stabilize the disulfide subloop. This may be the reason three enzymes have diverged away from the canonical E-class interface with large amino acid substitutions in place of human Gly150 and Ile151 (fig. 2*D*).

Genome and CuZnSOD coding sequence GC analysis was performed for all 34 proteins (supplementary figs. S8–S11, Supplementary Material online). A strong correlation between genome and CuZnSOD coding sequence GC content exists (supplementary fig. S12, Supplementary Material online) and is indicative that the CuZnSOD coding sequences are habituated to their genetic, metabolic, and environmental surroundings rather than being recent additions to their respective bacterial genomes.

### Metalation, Oligomeric State and Activity of Two Eukaryotic-Like Bacterial CuZnSODs

Of the CuZnSOD sequences described above, the least likely to form an E-class homodimer interface are those with bulky side-chain amino acid substitutions in place of human SOD1 Gly150 or those with a prokaryotic-like disulfide configuration. To investigate, representative Group 2 and 3a enzymes *Rb*CuZnSOD (OUV78940) and *Bb*CuZnSOD (OFX46851) were purified recombinantly from *E. coli*. This yielded zinc metalated proteins with negligible copper as is the case for human SOD1 (Wright et al. 2013). Both proteins were able to bind copper provided during purification at 4.4:1 and 0.8:1 Cu:Zn ratios for *Bb*CuZnSOD and *Rb*CuZnSOD, respectively. Nonstoichiometric *Bb*CuZnSOD copper binding is likely due to adventitious surface binding by His102-Glu104 and His102-Glu74-Asp39 sites as is the case when the protein is crystalized with zinc (7B4O). Addition of copper to the as-isolated protein does not however change the oligomeric state of the protein (supplementary fig. S13, Supplementary Material online) which has a molecular mass determined by size-exclusion chromatography coupled static light scattering (SEC-SLS) of 30.8 kDa (polydispersity index 1.01) against a predicted dimer mass of 32.4 kDa. This indicates *Bb*CuZnSOD forms a homodimer in solution.

**Fig. 3.**
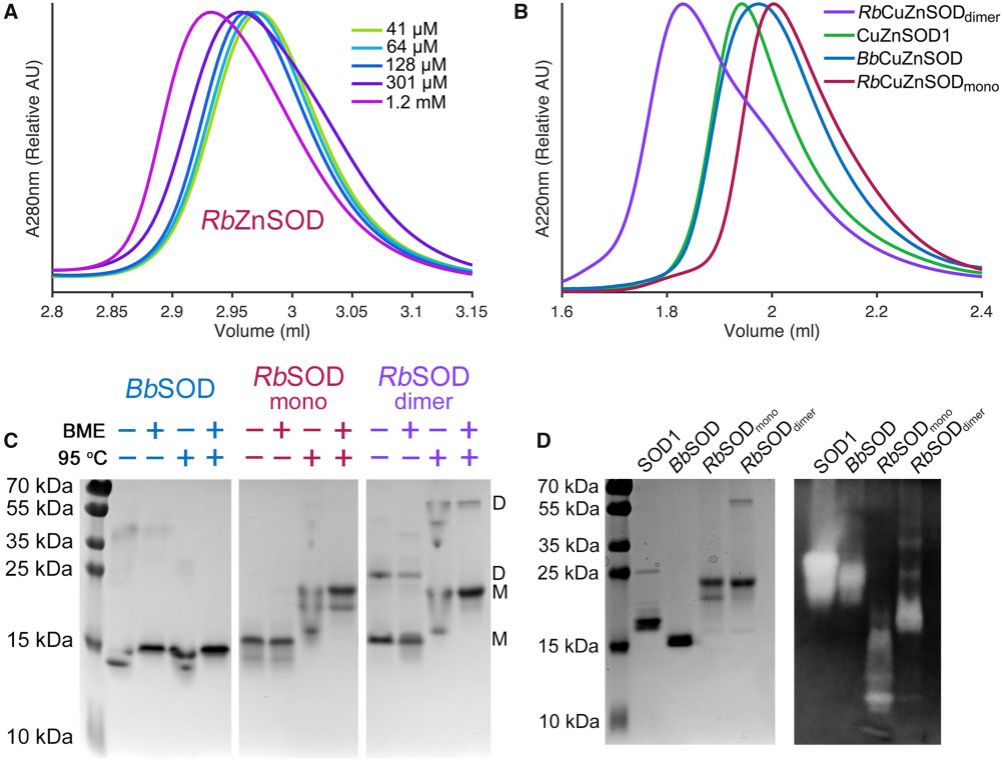
Oligomeric state and superoxide dismutase activity of *Rb*CuZnSOD and *Bb*CuZnSOD. (*A*) SEC chromatograms showing the increase in *Rb*ZnSOD hydrodynamic radius as concentration increases. (*B*) SEC chromatograms showing distinct *Rb*CuZnSOD monomers and dimers along with *Bb*CuZnSOD1 and SOD1dimers. (*C*) SDS-PAGE showing *Rb*CuZnSOD dimers are resistant to heating and reduction in contrast to *Bb*CuZnSOD. D—dimer, M—monomer, BME—β-mercaptoethanol. (*D*) Reducing and denaturing SDS-PAGE (left) of CuZnSODs assessed for superoxide dismutase activity by in-gel nitrotetrazolium blue staining (Beauchamp and Fridovich 1971). *Rb*CuZnSOD retains activity in the monomeric state.

*Rb*ZnSOD, in the zinc only metalated form, is predominantly monomeric and fails to fully dimerize even at millimolar concentrations (fig. 3*A*). On copper metalation, a species corresponding to dimeric *Rb*CuZnSOD could be separated from the monomeric form (fig. 3*B*). *Rb*CuZnSOD monomers and dimers have identical copper and zinc metalation ratios. Molecular masses calculated by SEC-SLS are 24.7 and 47.9 kDa (polydispersity indexes: 1.01 and 1.00) against expected masses from primary sequence of 19.7 and 39.5 kDa, respectively, for monomeric and dimeric species. Once formed, dimeric *Rb*CuZnSOD is exceptionally stable. Dissociation into monomers on SDS-PAGE is limited even when heated to 95 °C, the presence of reductant, or a combination of both (fig. 3*C*). *Bb*CuZnSOD and human SOD1 have similar mobility through size exclusion chromatography and native PAGE which, given their high sequence identity, similar isoelectric points (5.8 and 5.7, respectively) and solution molecular masses, is a good indication they adopt similar quaternary structure (fig. 3*B* and *D*). *Bb*CuZnSOD and *Rb*CuZnSOD are active superoxide dismutases (fig. 3*D*). Human SOD1 is known however to loose activity when monomeric (Banci et al. 1995) but *Rb*CuZnSOD monomers and dimers, which exhibit distinct mobility through native-PAGE, have equatable dismutase activity (fig. 3*D*).

### Intrasubunit Disulfide Formation

**Fig. 4.**
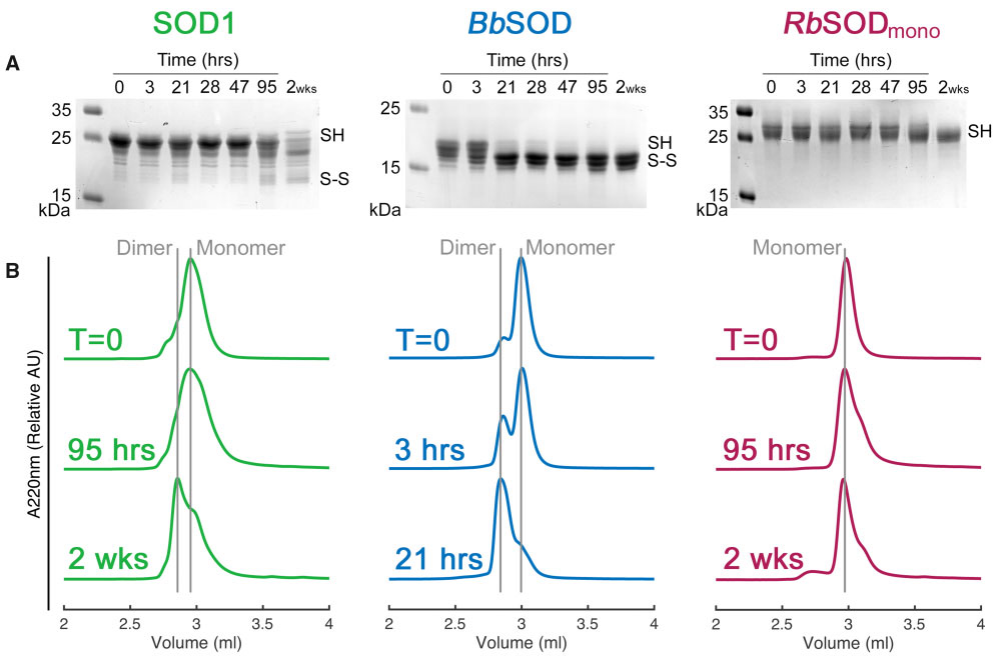
Intrasubunit disulfide bond formation and homodimerization. (*A*) Reducing and denaturing SDS-PAGE showing AMS-conjugated CuZnSODs following incubation over a time course. *Bb*ZnSOD is unreactive to AMS after 21 h showing free protein thiols have been sequestered indicating intrasubunit disulfide formation. In contrast 2 weeks is necessary for human SOD1 and *Rb*ZnSOD to display significant disulfide formation. (B) SEC chromatograms showing *Bb*ZnSOD homodimerization follows the same course as intrasubunit disulfide formation. Human SOD1 does eventually form homodimers while *Rb*ZnSOD remains monomeric over the same time period.

Formation of the human SOD1 intrasubunit disulfide bond promotes homodimerization by creating a stable interface region where intersubunit hydrogen bonding can take place. However, the SOD1 disulfide forms slowly when functional hCCS is not present and can be reduced by cellular reductants (Banci et al. 2012; Bouldin et al. 2012). Periplasmic bacterial CuZnSODs can follow a different route to maturation as exemplified by the CueP-SodC copper pathway of *S. enterica* (Osman et al. 2013) and DsbA-induced oxidative folding of *E. coli* CuZnSOD (Battistoni et al. 1999). Figure 4 indicates that *Bb*ZnSOD is able to form its intrasubunit disulfide bond rapidly in isolation in contrast to SOD1 and *Rb*ZnSOD. As the disulfide forms, *Bb*ZnSOD transitions from monomers to homodimers indicating *Bb*CuZnSOD forms an E-class homodimer through an interface incorporating the disulfide subloop and that it may follow an autonomous maturation pathway.

### Structure of *Bacteroidetes bacterium* E-Class CuZnSOD

**Fig. 5.**
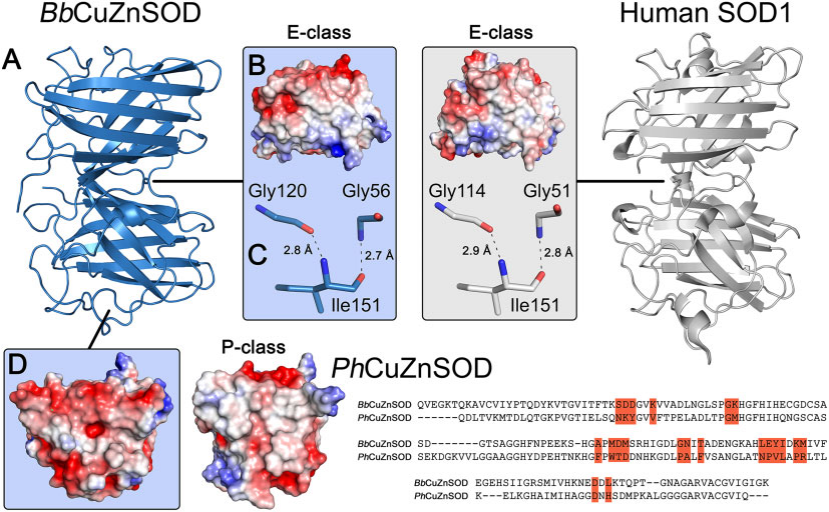
Bacterial *Bb*CuZnSOD dimerizes through an E-class interface. (*A*) Structure of homodimeric *Bb*CuZnSOD showing tertiary and quaternary structure similarity to human SOD1 (2C9V). (*B*) Surface charge map showing the *Bb*CuZnSOD interface is composed of a nonpolar patch which is conserved in human SOD1. (*C*) The *Bb*CuZnSOD interface maintains E-class intersubunit hydrogen bonding using the same amino acid type, bond lengths, and angles as human SOD1. (*D*) Surface charge map showing *Bb*CuZnSOD does not exhibit a nonpolar region which contributes to P-class homodimerization as exemplified by *Photobacterium leiognathi Ph*CuZnSOD (1YAI) and has very poor conservation (9% identity) of interface residues (highlighted orange).

To structurally characterize this new subdivision of eukaryotic-like bacterial superoxide dismutases, *Bb*CuZnSOD was crystallized and its structure determined in zinc-zinc and copper–zinc metalation states (supplementary fig. S14, Supplementary Material online) at 1.41 Å and 2.7 Å resolution, respectively. Crystallographic statistics are presented in supplementary table S10, Supplementary Material online. *Bb*CuZnSOD has a typical eight stranded, anti-parallel, Greek-key β-barrel core structure with two long loops incorporating conserved metal-binding ligands and coordination geometry (Richardson et al. 1975; Parge et al. 1992). Unusually, *Bb*CuZnSOD incorporates a β-barrel plug residue substitution reminiscent of the ALS-related Leu106Phe mutation (supplementary fig. S15*A*–*C*, Supplementary Material online). Comparison of *Bb*CuZnSOD monomers with human SOD1, *Ph*CuZnSOD and *E. coli* CuZnSOD monomers gives mean all-atom RMSD of 1.83 Å, 2.59 Å, and 4.22 Å, respectively. *Bb*CuZnSOD forms an E-class homodimer (fig. 5*A*) through a hydrophobic interface (fig. 5*B*) and maintains four symmetrical intersubunit hydrogen bonds typical of E-class eukaryotic CuZnSODs: Gly56-Ile151 2.7 Å and Ile151-Gly120 2.8 Å (fig. 5*C*). Interface hydrogen bond lengths are identical to those found in human SOD1 (fig. 5*C*). Strong hydrogen bonding is facilitated by conservation of Gly150 allowing close contact between opposing Gly51 and Ile151. There is no indication of a P-class interface (fig. 5*D*). *Bb*CuZnSOD is therefore sequentially (49.4% identity) and structurally more similar to human SOD1 than any bacterial P-class or P-class-like monomer CuZnSOD (32.7 ± 2.4% identity for those in the PDB), as predicted by phylogenetic analysis (fig. 1*C*).

**Fig. 6.**
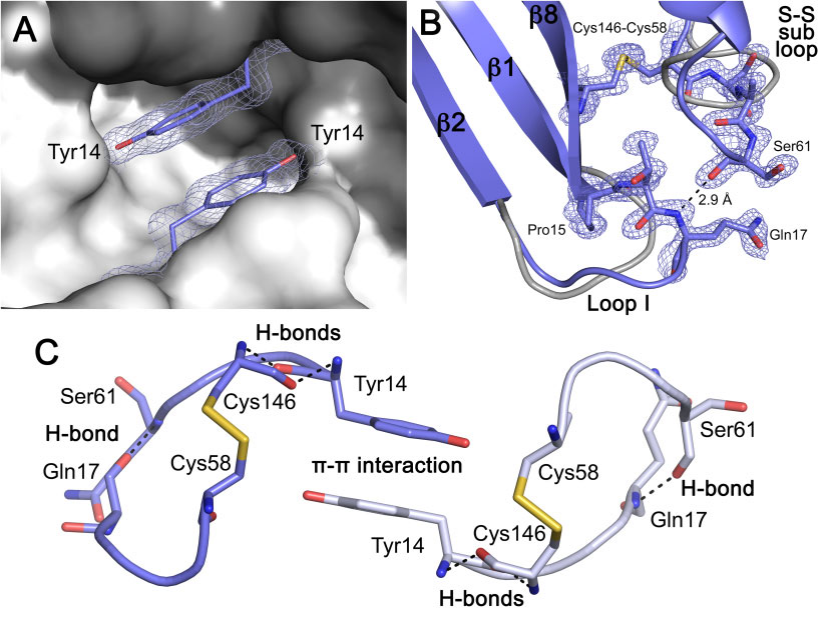
The *Bb*CuZnSOD disulfide subloop is stabilized by interactions with Try14 and loop I. (*A*) 2Fo-Fc electron density map contoured at 2σ showing opposing Tyr14 residues forming side-chain hydrophobic interactions (3.4 Å) at the *Bb*CuZnSOD homodimer interface aligned with the same site on the human SOD1 surface (gray and dark gray). (*B*) 2Fo-Fc electron density map contoured at 2σ showing how the *Bb*CuZnSOD disulfide subloop is stabilized by a hydrogen bond interaction with loop I Gln17. Both loops are longer than that found in human SOD1 (gray). (*C*) The *Bb*CuZnSOD interface interaction network. Reciprocal and symmetric interactions described in (*A*) and (*B*) combine to stabilize disulfide subloops and the homodimer interface. Residues from opposing monomers are colored blue and gray with hydrogen bonds dashed black.

The *Bb*CuZnSOD E-class dimer interface area is 747 Å^2^ comprising N-terminus, C-terminus, and disulfide subloop regions with a PISA (Krissinel and Henrick 2007) score of 1.0 as opposed to 0.2 and 0.3 for human and bovine SOD1, respectively. The *Bb*CuZnSOD interface is larger than both human and bovine SOD1 due to aromatic stacking of opposing Tyr14 side chains in a cavity at the dimer interface (fig. 6*A*). In addition, each Tyr14 forms two back-bone hydrogen bonds with disulfide bonding Cys146; β-barrel interactions that are conserved in human SOD1. Tyrosine is not common at this position in either eukaryotic CuZnSODs (0.5%) or the 34 bacterial enzymes presented in supplementary tables S1–S4, Supplementary Material online (9%) but occupation of this cavity with small molecules has been explored as a route to homodimer stabilization for SOD1-related ALS therapy (Ray et al. 2005; Wright et al. 2013). Immediately adjacent to Tyr14 at the loop I N-terminus, Pro15 replaces a glycine which is conserved in 80.5% of eukaryotic CuZnSODs (Gly10, human SOD1). As a result, loop I adopts a conformation shifted toward the disulfide subloop. Two extra amino acids are present in loop I allowing contact with the disulfide subloop and formation of a 2.9 Å backbone hydrogen bond between Gln17 amine and Ser61 carbonyl (fig. 6*B*). The disulfide subloop is itself extended by one amino acid to further accommodate this interaction. The lengths of both loops are conserved throughout the Group 3a and 3b bacterial CuZnSODs. This combination of interactions and extended loops creates a bridging network that spans the dimer interface to link intrasubunit disulfide bonds of opposing monomers (fig. 6*C*). This system appears to lend conformational restraint to the disulfide subloop prior to formation of the intrasubunit disulfide bond and may represent a mechanism for rapid disulfide formation (fig. 4).

### *BbCuZnSOD* Incorporates P-Class CuZnSOD Loop Features

**Fig. 7.**
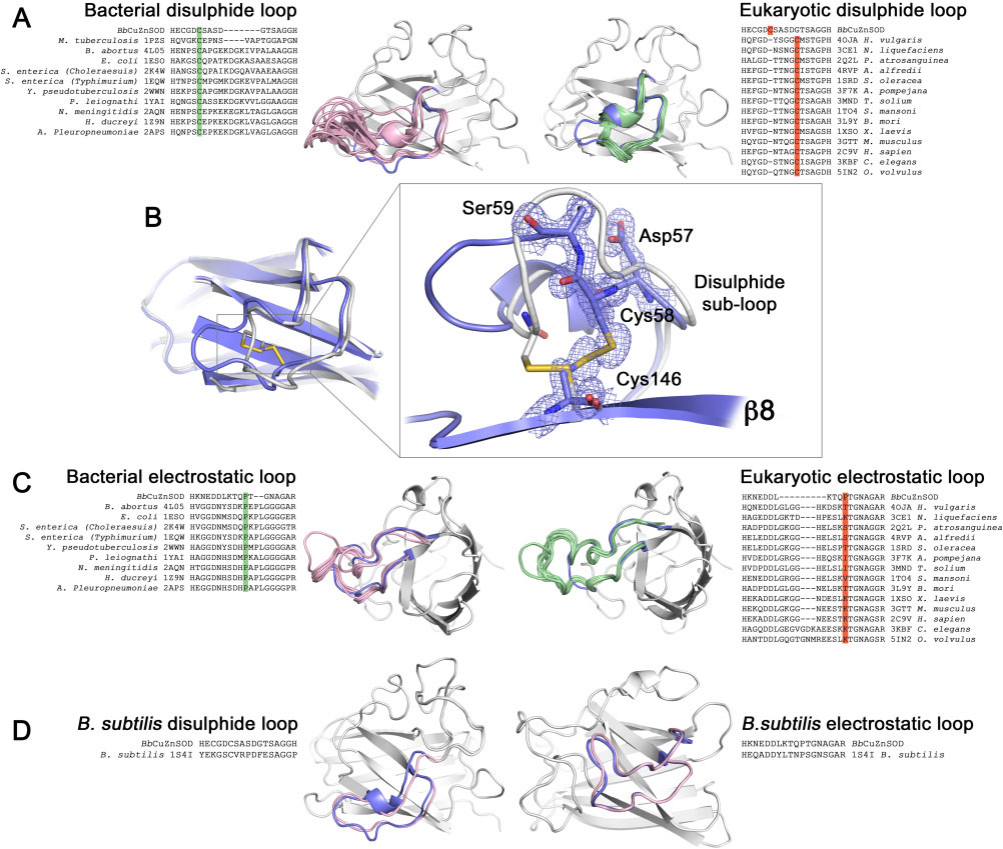
Eukaryotic-like *Bb*CuZnSOD β-barrel loops display similarities to P-class CuZnSODs. (*A*) Sequence and structural comparison of the *Bb*CuZnSOD disulfide subloop with analogous P-class and E-class CuZnSOD structures shows *Bb*CuZnSOD loops are of intermediate amino acid length and conformational extension. Structural features are highlighted pink—bacterial, green—eukaryotic, and blue—*Bb*CuZnSOD with all structures aligned to the *Bb*CuZnSOD β-barrel (gray). Sequence data show *Bb*CuZnSOD has a bacterial disulfide subloop cysteine configuration (green) as opposed to eukaryotic (orange). (B) 2Fo-Fc electron density map contoured at 2σ showing *Bb*CuZnSOD has a characteristically prokaryotic intrasubunit disulfide bond configuration (blue) in comparison with human SOD1 (gray). (*C*) Sequence and structural comparison of the *Bb*CuZnSOD electrostatic loop with bacterial and eukaryotic structures. The *Bb*CuZnSOD has length and conformation similar to bacterial orthologs with a characteristic and highly conserved central proline (green) that is absent in eukaryotic enzymes (orange). Structural features are colored as in (*A*). (*D*) Comparison of *Bb*CuZnSOD loop sequence and conformation with that of *B. subtilis* (1S4I). *Bb*CuZnSOD loop identity to *B. subtilis* is 47% and 56% in comparison with *Alvinella pompejana* 59% and 61% for disulfide subloop and electrostatic loops, respectively (gaps excluded). Structural features are colored as in (*A*).

Group 3a and 3b CuZnSODs form an E-class phylogenetic group between eukaryotic and P-class CuZnSODs (fig. 1*C*). However, P-class commonalities are discernable in these eukaryotic-like enzymes. P-class and P-class-like CuZnSODs have 20 or 24 amino acid disulfide subloops that extend into solvent while E-class dimers have compact 16 amino acid subloops (fig. 7*A*). *Bb*CuZnSOD, and all Group 3a and 3b enzymes with the exception of *Candidatus margulisbacteria* CuZnSOD (OGI05321), have an intermediate 17 amino acid disulfide loop (fig. 7*A*). In addition, P-class enzymes form Van der Waal’s and hydrophobic interactions between loop I and their disulfide suploop in a manner similar to *Bb*CuZnSOD (fig. 6*B*). Most importantly, all Group 3a enzymes, including *Bb*CuZnSOD, conform to a P-class intrasubunit disulfide configuration where the lower sub-loop links to β-strand 8 (figs. 2*C* and 7*B*).

A 20-amino acid electrostatic loop with a central proline is found in every P-class or P-class-like bacterial CuZnSOD characterized to date (fig. 7*C*). This enables a short turn around the zinc site often with no secondary structure. This feature is completely absent from all eukaryotic CuZnSODs present in the OMA database and the PDB which display 24 or 27 amino acid, α-helical electrostatic loops with no central proline (fig. 7*C*). The electrostatic loop proline is therefore a signal characteristic of prokaryotic CuZnSODs and is conserved in *Bb*CuZnSOD (Pro136) (fig. 7*C*) and across all Group 3a and 3b enzymes. As a result, the 18 amino acid *Bb*CuZnSOD electrostatic loop more closely resembles analogous structures found in P-class and P-class-like CuZnSODs than eukaryotic CuZnSODs (fig. 7*C*).

A single example illustrates the chimeric nature of Group 3a proteins well. The *Bb*CuZnSOD disulfide subloop and electrostatic loop have identical lengths and very similar conformations to those of a CuZnSOD-like protein of the gram-positive bacteria *Bacillus subtilis* (1S4I) (Banci et al. 2005) (fig. 7*D*) which is part of the P-class clade (fig. 1*C*). Despite this, *Bb*CuZnSOD shares higher loop identity with the eukaryotic extremophile *Alvinella pompejana* CuZnSOD (3F7K) which is structurally (Shin et al. 2009) and phylogenetically E-class (figs. 1*C* and 7*A* and *C*).

## Discussion

Superoxide dismutases have evolved independently at least three times throughout evolution. Each new iteration was cofactored by different redox metals as environmental conditions dictated available resources. CuZnSODs are present in all three domains of life: eukaryotes (McCord and Fridovich 1969; Goscin and Fridovich 1972; Sawada et al. 1972; Ferro et al. 2015), bacteria (Battistoni et al. 1996; Bourne et al. 1996; Forest et al. 2000; Pesce et al. 2000; Spagnolo et al. 2004; Mori et al. 2008; Pratt et al. 2015), and archaea (supplementary table S11, Supplementary Material online). All have the same metal-coordinating ligands in the same order within the primary structure indicating a monophyletic origin with metal binding and acquisition of activity predating evolution of eukaryotes (Forest et al. 2000). Despite a single origin, bacterial and eukaryotic CuZnSODs characterized to date differ considerably with respect to sequence, loop structures, and dimerization interface (Parge et al. 1992; Bourne et al. 1996). The Group 3 CuZnSODs described here and exemplified by *Bb*CuZnSOD represent an evolutionary intermediary between E-class and P-class enzymes with high eukaryotic sequence identity and E-class homodimerization but P-class functional loops and disulfide configuration. The global distribution of E-class bacterial CuZnSODs, their occurrence in many different phyla and adaptation of coding sequence GC content to their parent genomes indicate they are very likely to have been present at the time of eukaryogenesis. Despite exceptions within the protists and nematodes, which have lost antioxidant enzymes including CuZnSODs over time, E-class CuZnSODs are extremely well conserved across eukaryotes indicating they were present in the last common eukaryotic ancestor (McCord and Fridovich 1969; Goscin and Fridovich 1972; Sawada et al. 1972; Ferro et al. 2015). The absence of archaeal E-class CuZnSODs (supplementary table S11, Supplementary Material online) and the well described parallels between eukaryotic mitochondria and proteobacteria, particularly Rickettsiales species (Fan et al. 2020), indicate that an ancestral bacterial but eukaryotic-like CuZnSOD arrived in the proto-eukaryotic cell through endosymbiosis. That early eukaryotic CuZnSOD could be represented today by Group 1 or proteobacterial Group 2 enzymes with E-class disulfide and loop structures or Group 3a enzymes with remnant P-class features.

E-class CuZnSOD dimers have higher metal-binding affinity but lower activity than P-class dimers (Gabbianelli et al. 2004). This facilitates copper retention by intracellular enzymes but the E-class interface also allows engagement with a single, specific, and high-affinity cognate molecular, disulfide, and copper chaperone. CuZnSODs that have sufficiently high copper affinity to receive their cofactor from this metallochaperone distribution system allow maturation distant from the cytoplasmic membrane without reliance on metal ion diffusion (Pope et al. 2013) and within membrane-bound cellular compartments. The bacterial E-class CuZnSODs described here along with the copper-binding MXCXXC motif proteins typified by CopZ and found in all the bacterial phyla represented in supplementary table S5, Supplementary Material online are protein-domain building blocks requiring only duplication and shuffling to create a fully functional intracellular copper chaperone delivery system CuZnSODs resembling human and yeast CCS proteins. This system was in place before diversification of the last eukaryotic common ancestor where it facilitated the existence of large and organizationally complex cells within multicellular organisms.

## Materials and Methods

### Sequence Analysis

The eukaryotic CuZnSOD consensus sequence was determined by alignment of eukaryotic CuZnSOD orthologs downloaded from the OMA database (Altenhoff et al. 2018) in Jalview (Waterhouse et al. 2009). Putative eukaryotic-like bacterial CuZnSOD protein sequences were retrieved using the protein–protein BLAST (BLASTP) web interface and querying the eukaryotic CuZnSOD consensus sequence against the NCBI nonredundant (nr) database restricted to bacteria (taxid : 2) with default settings. Sequences were assessed for loosely conserved CuZnSOD disulfide subloop Gly-Asp-Gly-Cys and β-strand 1 and 2 sequences. Putative eukaryotic-like bacterial CuZnSOD sequences were then used to query the nr database with server BLASTP restricted to nonbacterial sequences. This yielded several protein sequences that had very high identity to a eukaryotic CuZnSOD including an annotated *Acinetobacter baumannii* protein (WP_071414557) with 97% identity to Scotts pine CuZnSOD (P24669). All were removed from subsequent analysis as they were likely due to sample contamination. Each putative bacterial CuZnSOD was also pair-wise aligned with human SOD1 and *Ph*CuZnSOD after removal of any signal peptide indicated by SignalP-5.0 (Almagro Armenteros et al. 2019). Transmembrane regions were predicted with TMHMM 2.0 (Möller et al. 2001). Identities are recorded in supplementary tables S1–S4, Supplementary Material online. Bacterial CuZnSODs were aligned with Muscle (Edgar 2004) and assigned to different groups based primary sequence motifs including disulfide cysteine positioning, signal peptide, and cysteine-rich N-terminus.

*Cyanobacterium bacterium* QH_1_48_107, *Rickettsiales bacterium* TMED131, and *Bacteroidetes bacterium* GWA2_30_7 WGS sequencing data (PXPJ00000000, NHGG00000000, and MENC00000000, respectively) were separated into individual contigs and used as queries against the NCBI reference sequence (RefSeq) database (O’Leary et al. 2016) restricted to nonbacterial sequences using standalone BLASTX 2.10.0+ (Altschul et al. 1997). For each contig, a protein translation with the highest BLAST score to a nonbacterial protein sequence was used to query the RefSeq database restricted to bacterial proteins using server BLASTP. Results were filtered to remove proteins from organisms sequenced as part of the same study. The difference between BLAST scores for bacterial and nonbacterial sequences were then used to assess the likelihood genomic assemblies contained nonbacterial material. The top hit from each contig is recorded in supplementary datasets 1–3, Supplementary Material online. Translations of coding sequences immediately adjacent to *Cb*CuZnSOD, *Rb*CuZnSOD, and *Bb*CuZnSOD coding sequences were also used to interrogate the RefSeq database using BLASTP restricted to bacterial or nonbacterial sequences (supplementary tables S7–S9, Supplementary Material online, respectively). Genome GC content was analyzed using the Matlab ntdensity function.

### Phylogenetic Tree Building

To limit the effects of genetic saturation, protein sequences of every CuZnSOD found in the PDB and those listed in supplementary tables S1–S4, Supplementary Material online were aligned with Muscle (Edgar 2004) following removal of any signal peptide. The full alignment of 63 taxons and 332 characters was trimmed with Gblocks (Talavera and Castresana 2007) to remove divergent, misaligned, or gapped positions using both strict and loose parameters yielding alignments of 34 and 96 characters, respectively. An alignment trimmed to incorporate only the CuZnSOD β-barrel with internal loops and minimize gaps was created manually in Jalview (Waterhouse et al. 2009). Selection of best fit amino acid substitution rate models was performed for each alignment using the Akaike information criterion implemented in PhyML 3.0 (Guindon et al. 2010) and MEGA 10.8 (Stecher et al. 2020). Dayhoff (Dayhoff et al. 1978), Jones–Taylor–Thornton (JTT) (Jones et al. 1992) and Whelan–Goldman (WAG) (Whelan and Goldman 2001) fixed rate models were predicted with roughly equal validity. Phylogenetic analysis was performed by Bayesian inference with MrBayes 3.2 (Ronquist et al. 2012) with a gamma-distributed rate variation across all sites (G), a proportion of invariant sites combined with a gamma distribution for other sites (invG), and an invariant distribution across all sites (inv) for all alignments described above. Phylogenetic trees were also constructed using Le-Gascuel (LG), Blosum, and rtRev fixed rate models and GTR and Equalin variable rate models. Tree building was performed for 38 different parameter or alignment sets until the average standard deviation of split frequencies between two independent runs of four Markov Chain Monte Carlo (MCMC) chains was less than 0.005. The first 25% of sampled trees were discarded during analysis. P-class/P-class-like sequences and E-class/E-class-like sequences were separated into distinct clades for every tree constructed with 100% probability. The maximum likelihood method was also used to independently verify phylogenetic tree form using PhyML 3.0 (Guindon et al. 2010) using the WAG model. Unrooted trees were drawn with FigTree 1.4.4.

### Protein Expression and Purification

*Rickettsiales bacterium* TMED131 *Rb*CuZnSOD and *Bacteroidetes bacterium* GWA2_30_7 *Bb*CuZnSOD coding DNA was synthesized by Twist Bioscience along with an N-terminal hexa-histidine and SUMO tags and ligated into pET21 expression vector. Both proteins were expressed in BL21 (DE3) *E. coli* in LB media at 18 °C for 16 h with shaking at 180 rpm following induction with 0.4 mM isopropyl β-d-1-thiogalactopyranoside and 200 µM ZnCl_2_. Cells were lysed by sonication in 20 mM tris(hydroxymethyl)aminomethane-HCl pH 7.4, 500 mM NaCl, 20 mM imidazole, bound to Nickel-nitrilotriacetic acid resin and eluted with the same buffer including 500 mM imidazole. The His-SUMO tag was removed with SUMO protease in dialysis at 4 °C for 16 h against 20 mM tris(hydroxymethyl)aminomethane-HCl pH 7.4, 150 mM NaCl to yield the native protein sequence with no additional N-terminal amino acids. The protein was then concentrated and separated from remaining impurities on a Superdex S200 16 600 SEC column. Protein concentration was quantified by Bradford assay. Copper metalated protein was produced by dialysis against 20 mM tris(hydroxymethyl)aminomethane-HCl pH 7.4, 150 mM NaCl, 2 mM CuCl_2_ followed by 3-fold dialysis overnight against the same buffer without copper prior to preparative SEC. Samples for inductively coupled plasma metal analysis were prepared by incubation in 70% nitric acid for 24 h at room temperature before to dilution to 4.9% nitric acid.

### Analytical SEC and Molecular Mass Determination

An Agilent BioSEC Advance 300 Å, 4.6 × 300 mm or GE Superdex 200 5 15 column was used for analytical SEC and SEC-SLS in 20 mM tris(hydroxymethyl)aminomethane-HCl pH 7.4, 150 mM NaCl buffer. Both were performed on an Agilent 1260 Infinity Multi-Detector System using refractive index to calculate masses.

### In-Gel Superoxide Dismutase Activity

CuZnSOD activity was tested in gel according to an established protocol (Beauchamp and Fridovich 1971) where superoxide dismutase activity inhibits formation of blue formazan from nitrotetrazolium blue by superoxide generated by the photoreduction of riboflavin thereby creating colorless bands on a dark blue gel background. Briefly, 750 ng of protein was loaded on a 12% acrylamide nonreducing native PAGE gel and run at 100 V until the dye front left the gel. The gel was then washed in 50 mM K_2_HPO_4_ pH 7.5 for 10 min; 480 µM nitrotetrazolium blue chloride, 50 mM K_2_HPO_4_ pH 7.5 for 20 min, 30 µM riboflavin, 0.02% N,N,N′,N′-tetramethylethylenediamine, 50 mM K_2_HPO_4_ pH 7.5 for 20 min. The gel was then exposed to white light until banding became clear. About 1.25 μg of protein from the same samples, with the addition of 2-mercaptoethanol and heating at 95 °C for 5 min, was also separated by reducing and denaturing SDS-PAGE and stained by Coomasie blue.

### Disulfide Formation Assay

Zinc-bound, copper-free, disulfide-reduced proteins were produced by incubation of as-isolated superoxide dismutases with 40 mM dithiothreitol at 4 °C overnight. Reductant was removed by desalting into nitrogen-purged 20 mM tris(hydroxymethyl)aminomethane-HCl pH 7.4, 150 mM NaCl with PD Mini-tap G25 columns pre-equilibrated with buffer. Eluted proteins were diluted to 20 µM with nitrogen-purged buffer and incubated at room temperature for the experiment time course. Samples were taken periodically and analyzed by analytical SEC, as described above, or incubated with 400 µM 4-acetamido-4′-maleimidylstilbene-2,2′-disulfonic acid (AMS) for 90 min at 37 °C prior to separation by denaturing and reducing 15% acrylamide SDS-PAGE.

### Crystallization

As isolated *Bb*ZnSOD, lacking catalytic copper, was crystallized at 16 mg/ml using the sitting drop vapor diffusion method in 100 mM tris(hydroxymethyl)aminomethane-HCl pH 8, 20% polyethylene glycol 6000, 2 mM ZnCl_2_ at 22 °C for approximately 4 weeks. Crystals were cryoprotected in 100 mM tris(hydroxymethyl)aminomethane-HCl pH 8, 20% polyethylene glycol 6000, 20% glycerol, and flash frozen in liquid nitrogen. *Bb*CuZnSOD was crystallized as above but in Morpheus (Molecular Dimensions) screen condition 1-44 comprising: 0.12 M alcohol mix containing 1,6-hexanediol, 1-butanol 1,2-propanediol, 2-propanol, 4-butanediol, 1,3-propanediol; 0.1 M buffer mix containing sodium 4-(2-hydroxyethyl)-1-piperazineethanesulfonic acid, 3-(N-morpholino)propane sulfonic acid pH 7.5; 37.5% precipitant mix containing 2-Methyl-2,4-pentanediol, polyethylene glycol 1000, polyethylene glycol 3350, and flash frozen in the drop solution.

### Crystallographic Data Collection and Structure Solution

Diffraction data was collected at Synchrotron Soleil on beamline Proxima 1 at wavelength 0.98, 1.22, and 1.33 Å where appropriate. Data were indexed and integrated with XDS (Kabsch 2010) or iMosflm (Battye et al. 2011); scaled with Aimless within CCP4 (Winn et al. 2011); solved by molecular replacement using the human SOD1 structure (2C9V) as a search model, or the zinc-metalated *Bb*ZnSOD structure for the Cu-Zn metalated variant, with Molrep (Vagin and Teplyakov 1997); rebuilt with ARP/wARP (Langer et al. 2008, p. 7); and refined with Refmac (Murshudov et al. 2011, p. 5) and Coot (Emsley et al. 2010). Structures were validated with Molprobity and the PDB validation tool and deposited in the PDB with accession codes 7B4O and 7B4P. Electron density maps were created with Phenix Maps (Liebschner et al. 2019) and Pymol.

## Supplementary Material

Supplementary data are available at *Molecular Biology and Evolution* online.

## Supplementary Material

msab157_Supplementary_Data
