# Supplementary material for: Bacterial Evolutionary Precursors of Eukaryotic Copper–Zinc Superoxide Dismutases
Source: Mol Biol Evol. 2021 May 22;38(9):3789–803. doi: 10.1093/molbev/msab157 (PMC8382915; doi:10.1093/molbev/msab157)
Supplement: msab157_Supplementary_Data [file msab157_supplementary_data.zip › Bacterial E-type SOD - Supplementary.pdf]

Supplementary Information for:

Bacterial evolutionary precursors of eukaryotic copper-zinc superoxide dismutases

This file includes:

Figures S1 to S15
Tables S1 to S11
Legends for Datasets S1 to S3

Other supplementary materials for this manuscript:

Supplementary dataset 1 - Cyanobacterium bacterium QH_1_48_107.xlsx

Comparison of the similarity of Cyanobacterium bacterium QH_1_48_107 translated proteins with other bacterial proteins and non-bacterial proteins. The contig containing *Cb*CuZnSOD coding sequence is highlighted in blue.

Supplementary dataset 2 - Rickettsiales bacterium TMED131.xlsx

Comparison of the similarity of Rickettsiales bacterium TMED131 translated proteins with other bacterial proteins and non-bacterial proteins. The contig containing *Rb*CuZnSOD coding sequence is highlighted in blue.

Supplementary dataset 3 - Bacteroidetes bacterium GWA2_30_7.xlsx

Comparison of the similarity of Bacteroidetes bacterium GWA2_30_7 translated proteins with other bacterial proteins and non-bacterial proteins. The contig containing *Bb*CuZnSOD coding sequence is highlighted in blue.

Supplementary Figures

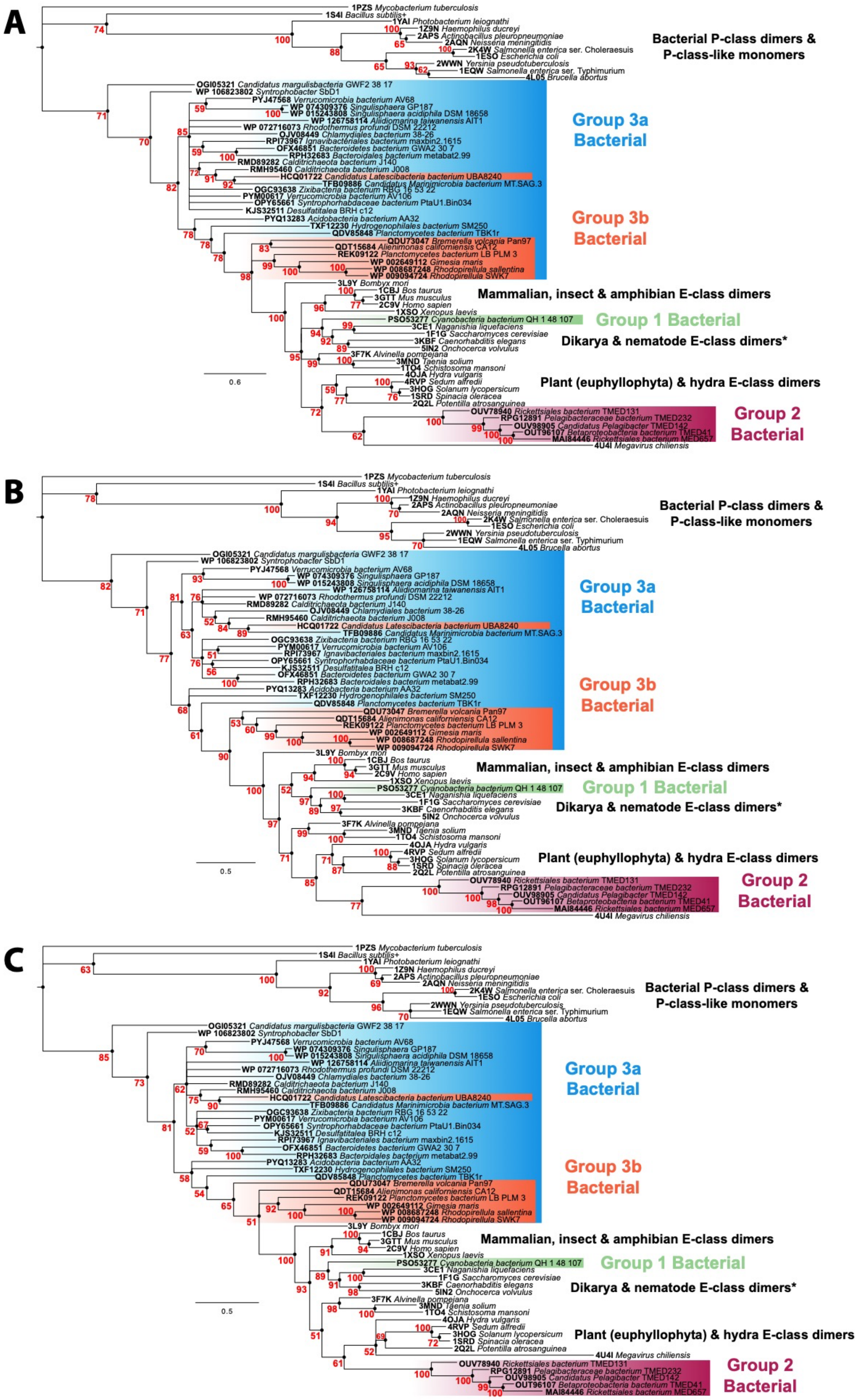

Fig. S1. Unrooted, 50% consensus majority-rule CuZnSOD phylogenetic trees constructed by Bayesian inference using different amino acid substitution models. Each tree was constructed using a complete 63 taxon, 332 character multiple sequence alignment of CuZnSODs presented in Table S1, S2, S3

and S4 and the PDB. *Megavirus chiliensis* and the P-class/P-class-like clade display long-branch attraction likely due to substitution rate heterogeneity and ancestral divergence respectively. The latter is common when comparing bacterial and eukaryotic proteins and makes assignment of divergence times directly from sequence data difficult but is unlikely to affect the segregation between eukaryotic/eukaryotic-like and P-class/P-class-like lineages which occurs with 100% probability for every tree. Branch lengths represent expected substitutions per site with scale bar at the bottom left of each tree. Numbers in red are posterior probability clade support values as a percentage. (**A**) Jones-Taylor-Thornton model (JTT) + G, 10.2 million generations, 30,722 sample size, potential scale reduction factor (PSRF) 1.000052, minimum effective sample size (mESS) 1520. (**B**) Dayhoff model +G, 12.4 million generations, 37,232 sample size, PSRF 1.000038, mESS 2945. (**C**) Whelan-Goldman model + G, 44.2 million generations, 132,542 sample size, PSRF 0.9999954, mESS 6384. * *C. elegans* CuZnSOD (3KBF) is monomeric. + Structure 1S4I from *B. subtilis* is not an active superoxide dismutase but retains a CuZnSOD-like structure.

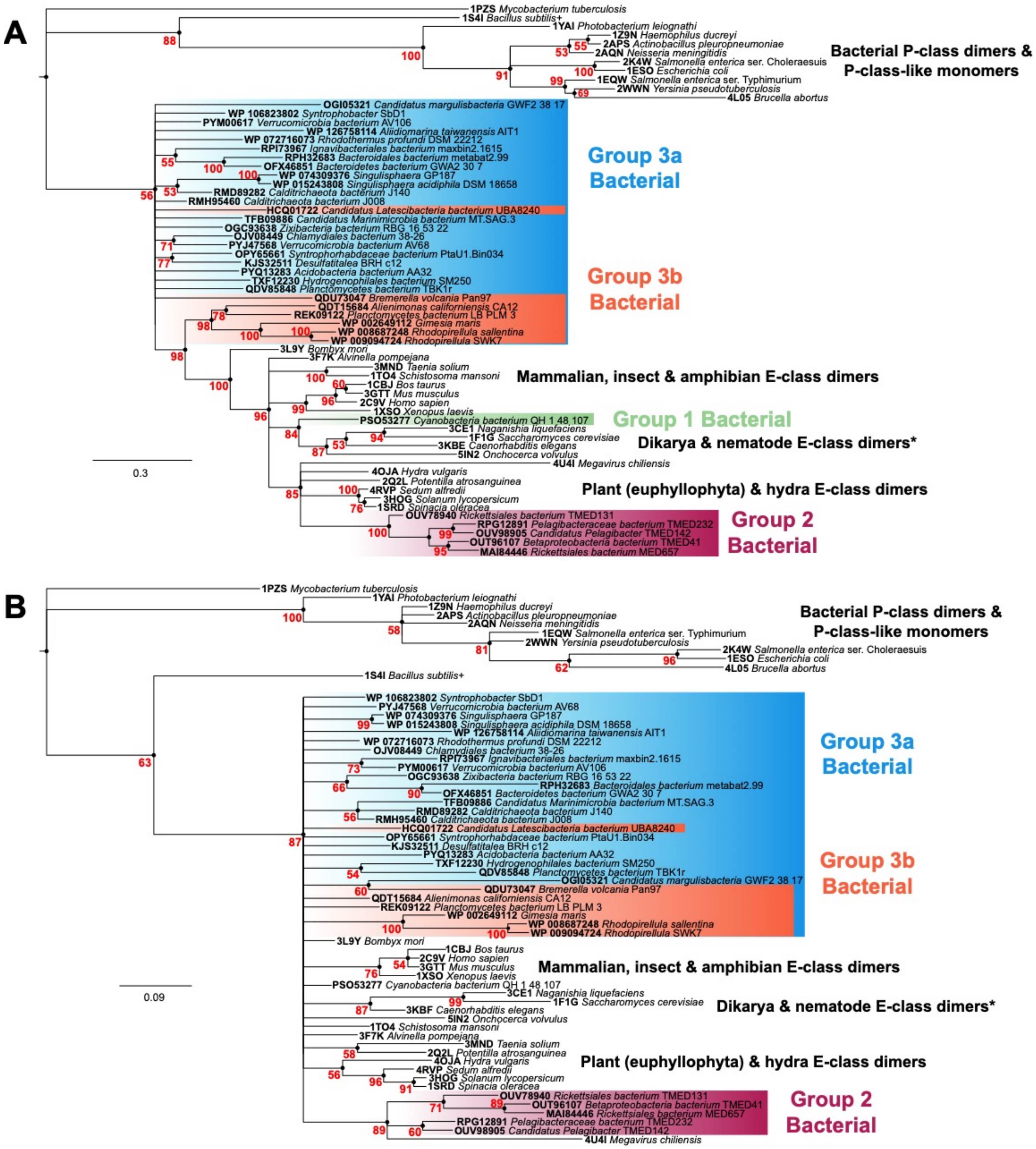

Fig. S2. Unrooted, 50% consensus majority-rule CuZnSOD phylogenetic trees constructed by Bayesian inference using JTT+G amino acid substitution model and trimmed multiple sequence alignment. Trees were constructed using 63 taxon alignments of (**A**) 96 characters and (**B**) 34 characters. Both phylogenetic trees have overall structure similar to those presented in Fig. 1 C and Fig. S1 however information loss as a result of removing less well conserved regions progressively compacts the E-class clade. Branch lengths represent expected substitutions per site with scale bar at the bottom left of each tree. Numbers in red are posterior probability clade support values as a percentage. (**A**) 16.6 million generations, 49,742 samples, PSRF 0.9999866, mESS 8807. (**B**) 22.5 million generations, 67,442 samples, PSRF 1.000034, mESS 5247. * *C. elegans* CuZnSOD (3KBF) is monomeric. + Structure 1S4I of *B. subtilis* is not an active superoxide dismutase but retains a CuZnSOD-like structure.

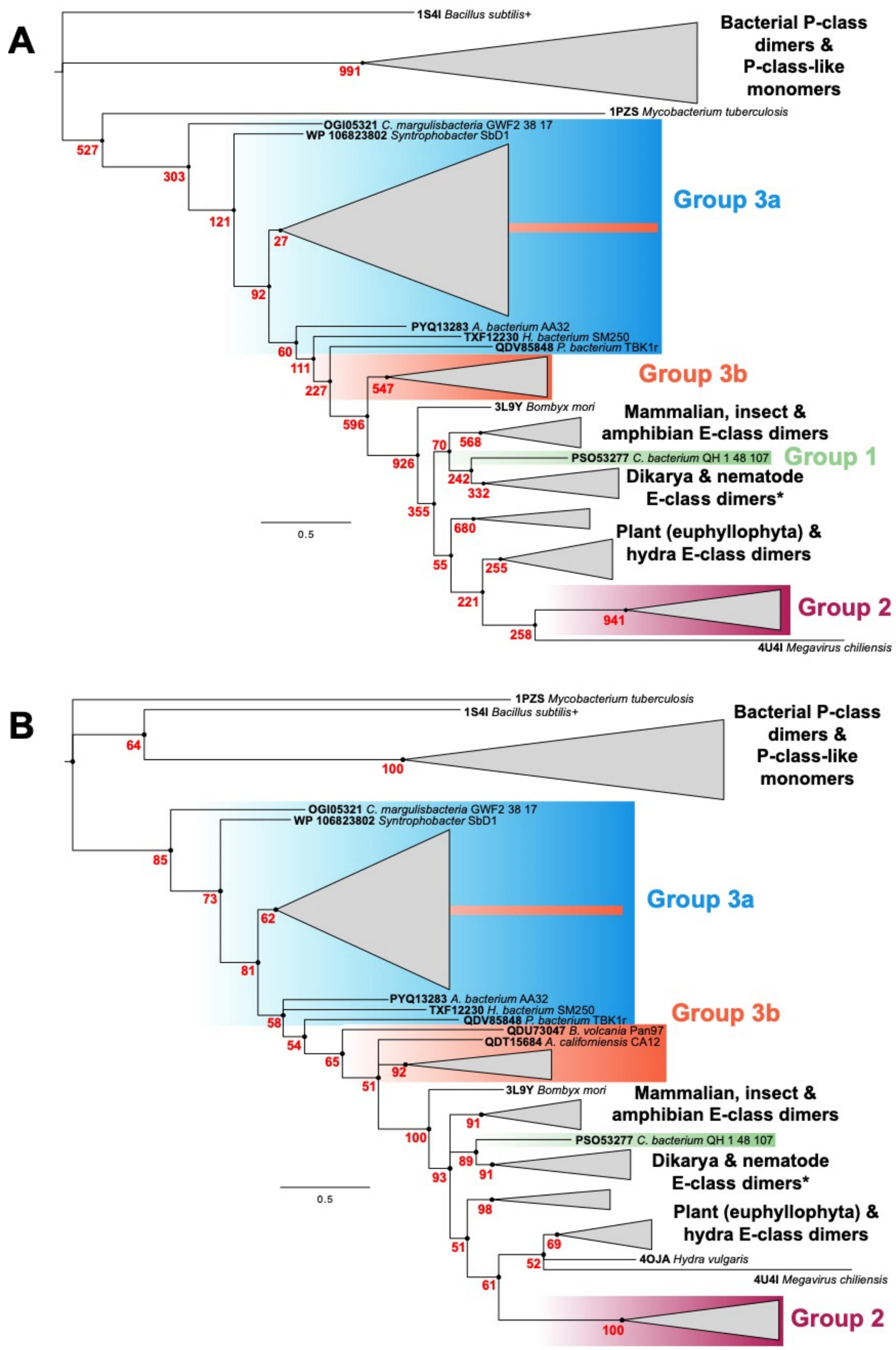

Fig. S3. Comparison of maximum likelihood and Bayesian inference tree construction methodologies for CuZnSODs. (**A**) Maximum likelihood phylogenetic tree using the WAG+invG substitution model, 10 initial trees were created with BIONJ and optimised with sub-tree pruning and re-joining. 1000 replicate bootstrapping branch support values are in red (**B**) Comparable Bayesian inference WAG + invG tree taken from Fig. S1 C with posterior probabilities in red. Both trees were generated from a full multiple sequence alignment of 332 characters and are representative of all trees generated with shorter alignments limited to more conserved positions. Branch lengths represent expected substitutions per site with scale bar at the bottom left of each tree. All terminal branches are compacted. * *C. elegans* CuZnSOD (3KBF) is monomeric. + Structure 1S4I of *B. subtilis* is not an active superoxide dismutase but retains a CuZnSOD-like structure.

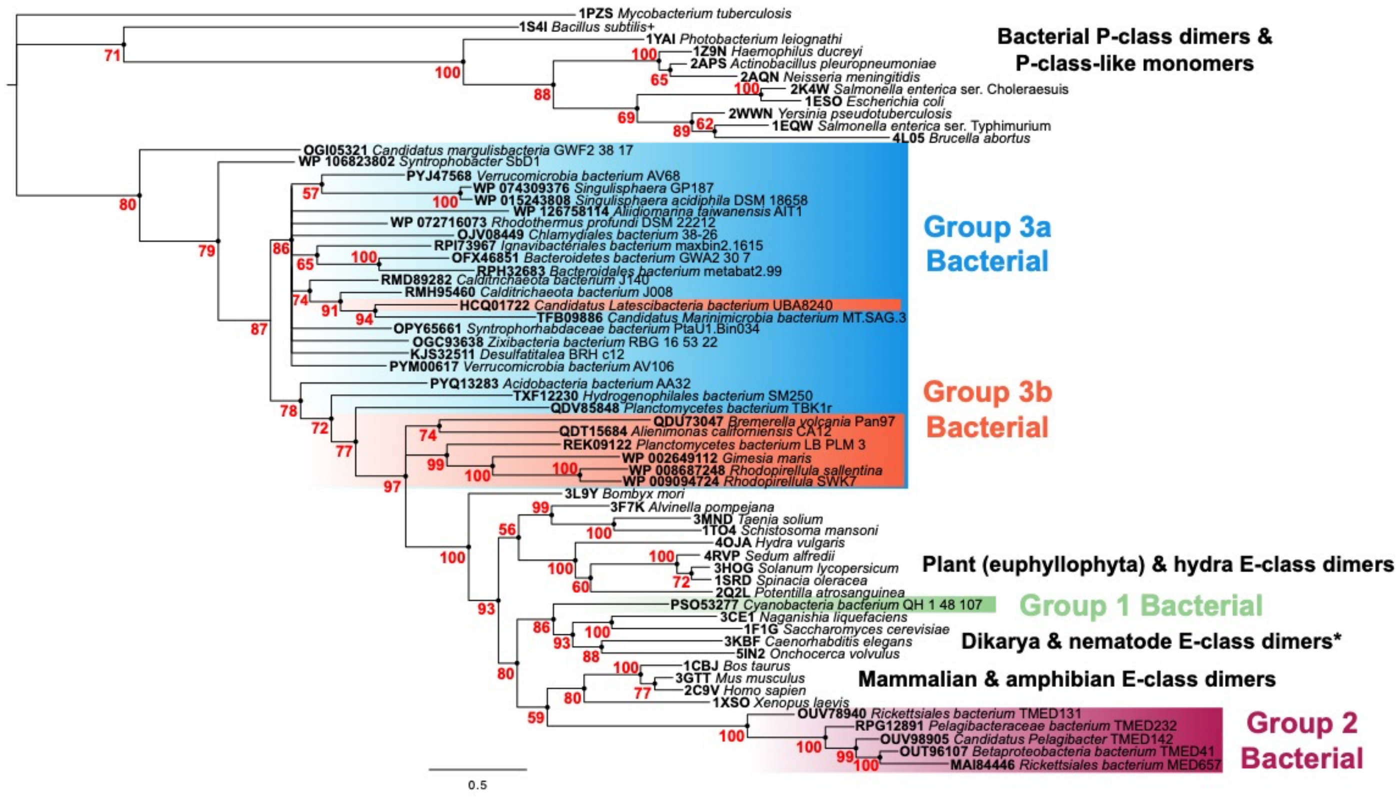

Fig. S4. Unrooted, 50% consensus majority-rule CuZnSOD phylogenetic tree constructed by Bayesian inference using JTT+G amino acid substitution model without Megavirus chiliensis CuZnSOD sequence. Phylogenetic trees presented in Fig. 1 C, S1, S2 and S3 show variability in the positioning of Megavirus chiliensis CuZnSOD. This sequence was removed to yield a 62 taxon, 332 character alignment. No significant differences in overall tree topology are observed. Branch lengths represent expected substitutions per site with scale bar at the bottom left. Numbers in red are posterior probability clade support values as a percentage. The tree is presented in full. 17.2 million generations, 51602 samples, PSRF 1.000111, mESS 6369. * C. elegans CuZnSOD (3KBF) is monomeric. + Structure 1S4I from B. subtilis is not an active superoxide dismutase but retains a CuZnSOD-like structure.

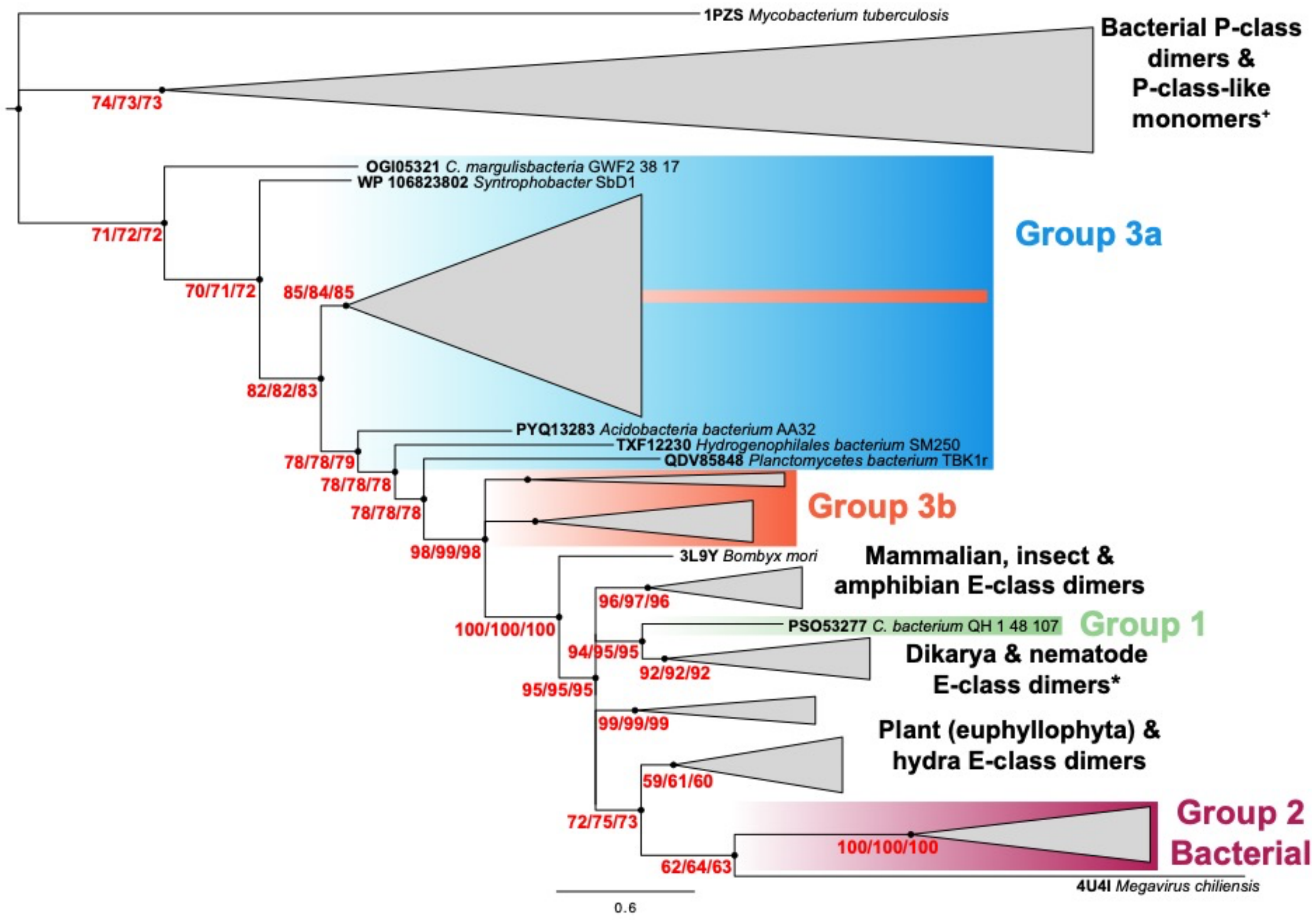

Fig. S5. Unrooted, 50% consensus majority-rule CuZnSOD phylogenetic tree constructed by Bayesian inference using JTT amino acid substitution model with different rate variation parameters. Trees were constructed from the full-length alignment. Posterior probability node support values in red are presented for three analyses: First, JJT+G (Fig. S1A), 10.2 million generations, 30,722 sample size, PSRF 1.000052, mESS 1520. Second, JJT+invG, 11.3 million generations, 33782 samples, PSRF 0.9999774, mESS 2009. Third, JJT+inv, 35.5 million generations, 106,532 samples, PSRF 1.000214, mESS 5354. Branch lengths represent expected substitutions per site with scale bar at the bottom of the tree. All terminal branches are compacted. * *C. elegans* CuZnSOD (3KBF) is monomeric. + Structure 1S4I of *B. subtilis* is not an active superoxide dismutase but retains a CuZnSOD-like structure.

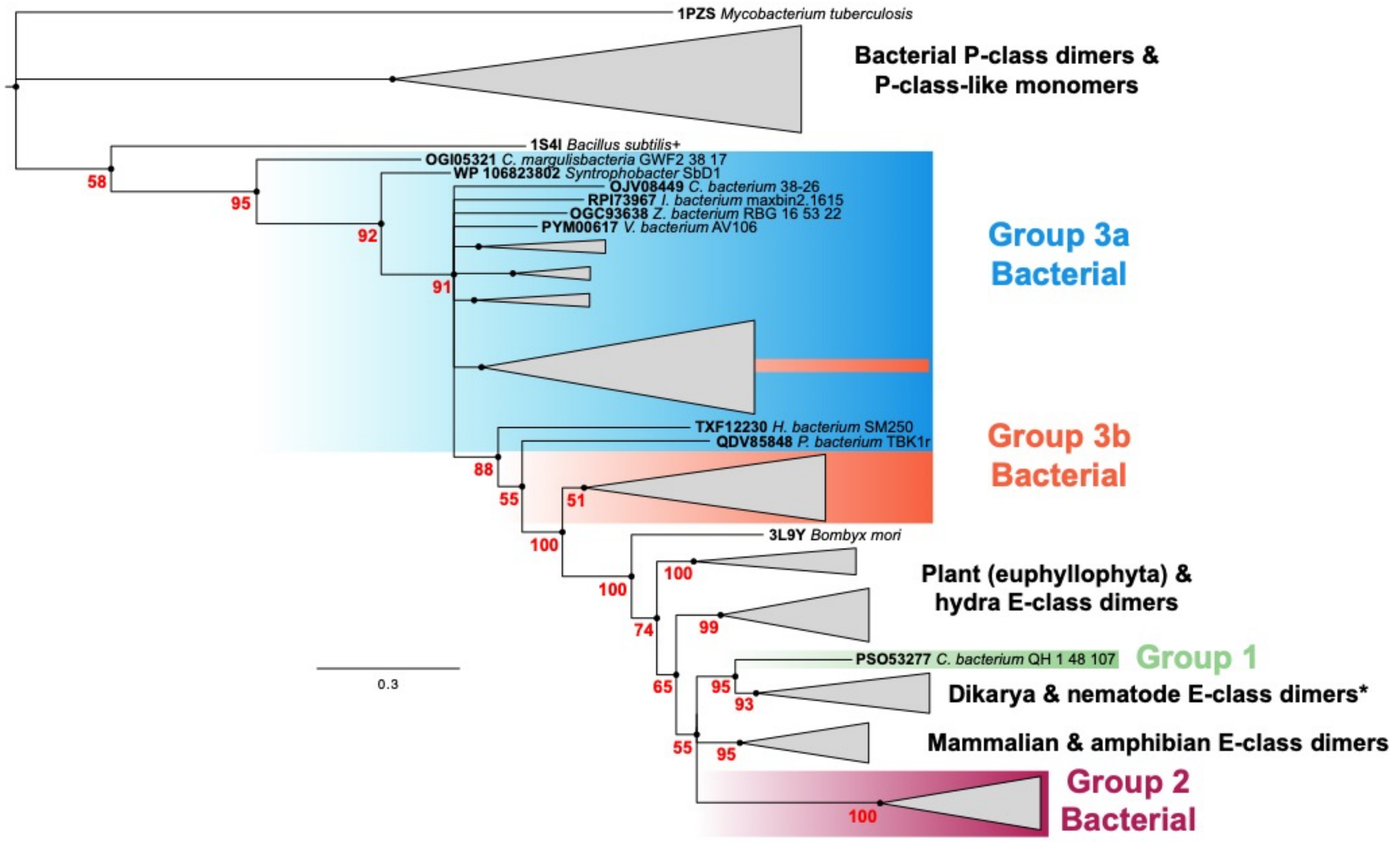

Fig. S6. Unrooted, CuZnSOD phylogenetic tree constructed from a manually trimmed CuZnSOD multiple sequence alignment. Multiple sequence alignment was restricted to the core CuZnSOD β-barrel with internal loops but with minimal gaps aligned without the Megavirus chiliensis sequence: 62 taxons, 142 characters. JJT+G, 49.8 million generations, 14942 samples, PSRF 0.9999702, mESS 4573. Branch lengths represent expected substitutions per site with scale bar at the bottom left. Numbers in red are posterior probability clade support values as a percentage.

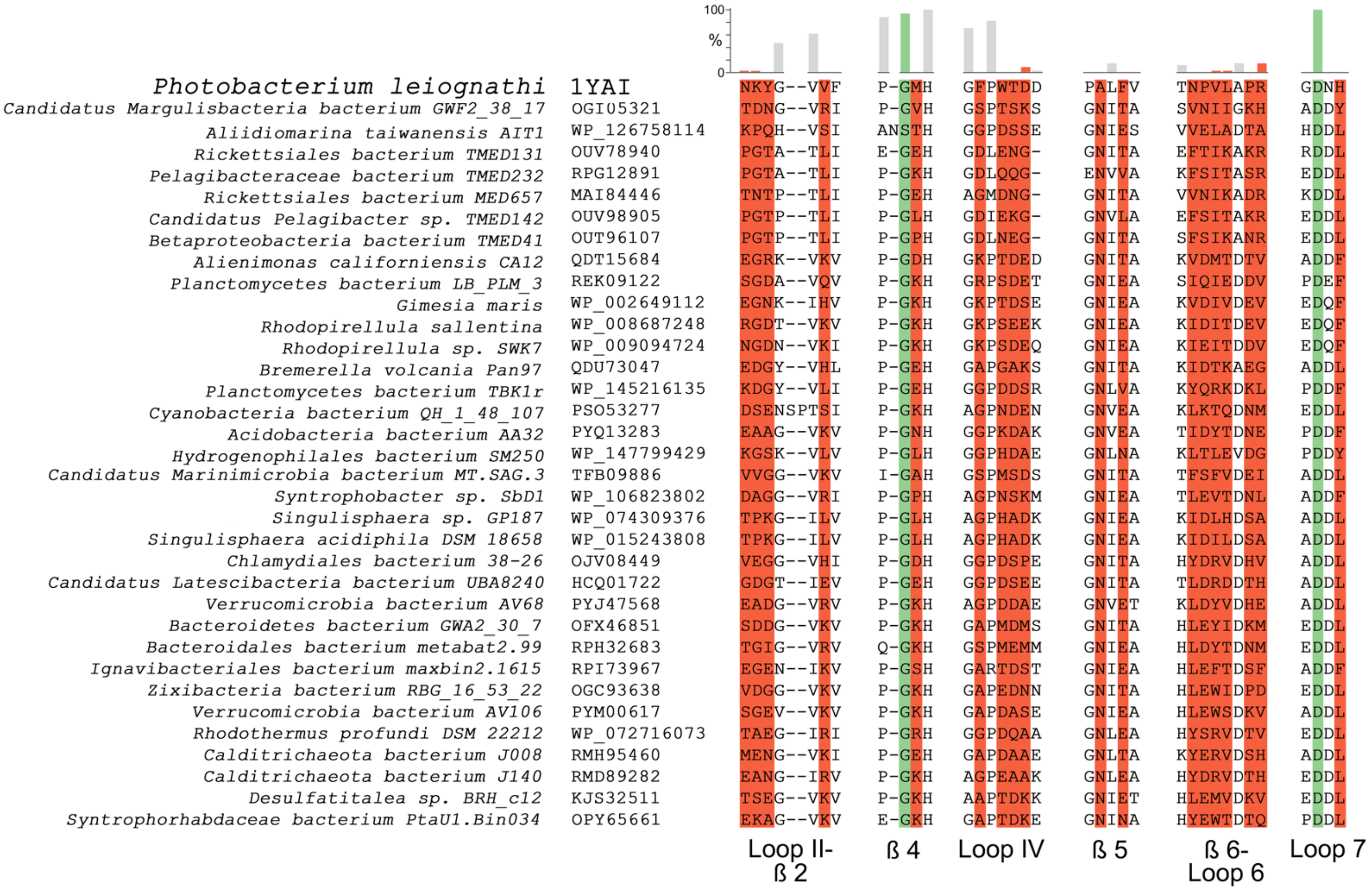

Fig. S7. Eukaryotic-like bacterial CuZnSODs do not conserve P-class homodimerisation interfaces. Interface residues are highlighted green and orange to indicate high and low conservation respectively compared with *P. leiognathi* CuZnSOD. Bar charts represent sequence conservation with those highlighted green and orange playing a role in *P. leiognathi* CuZnSOD homodimerisation.

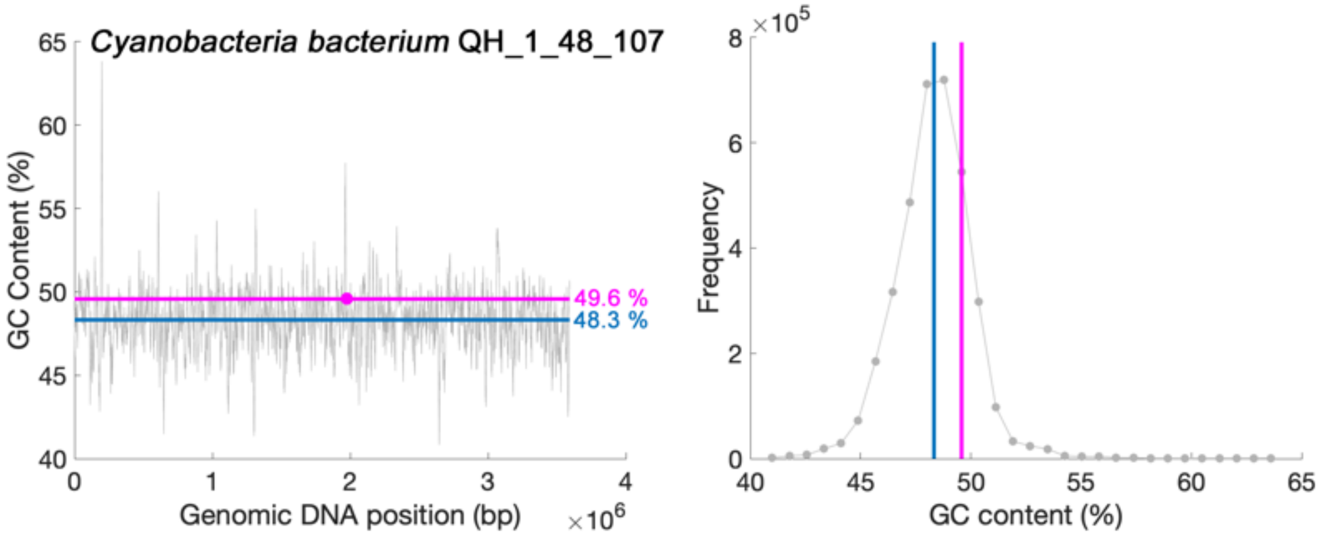

Fig. S8. Parent genome and CuZnSOD coding sequence GC content analysis for Group 1 enzyme *Cb*CuZnSOD (PSO53277). Left pane shows *Cyanobacterium bacterium* QH_1_48_107 genomic DNA GC content calculated as a moving window average over the whole sequence (grey), total genomic GC content (teal) and GC content of the *Cb*CuZnSOD coding sequence (pink). Right pane shows the GC content distribution for genomic fragments of size approximately equal to that of the *Cb*CuZnSOD (grey) along with whole genome (teal) and coding sequence (pink) GC content.

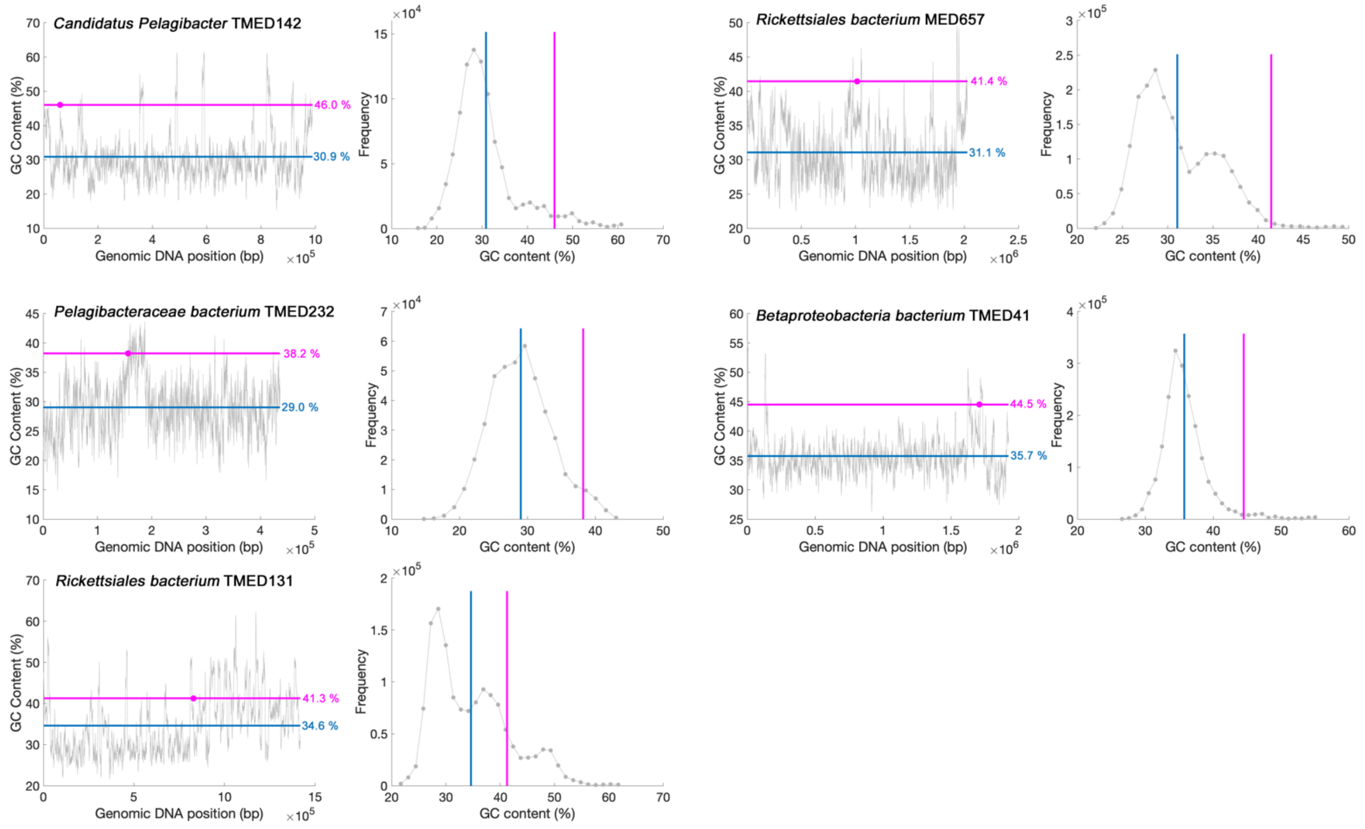

Fig. S9. Genome and CuZnSOD coding sequence GC content analysis for Group 2 CuZnSODs. Left panes show genomic DNA GC content calculated as a moving window average over the whole sequence (grey), total genomic GC content (teal) and GC content of the CuZnSOD coding sequence (pink). Right panes show the GC content distribution for genomic fragments of size approximately equal to that of the CuZnSOD coding sequence (grey) along with whole genome (teal) and CuZnSOD coding sequence (pink) GC content.

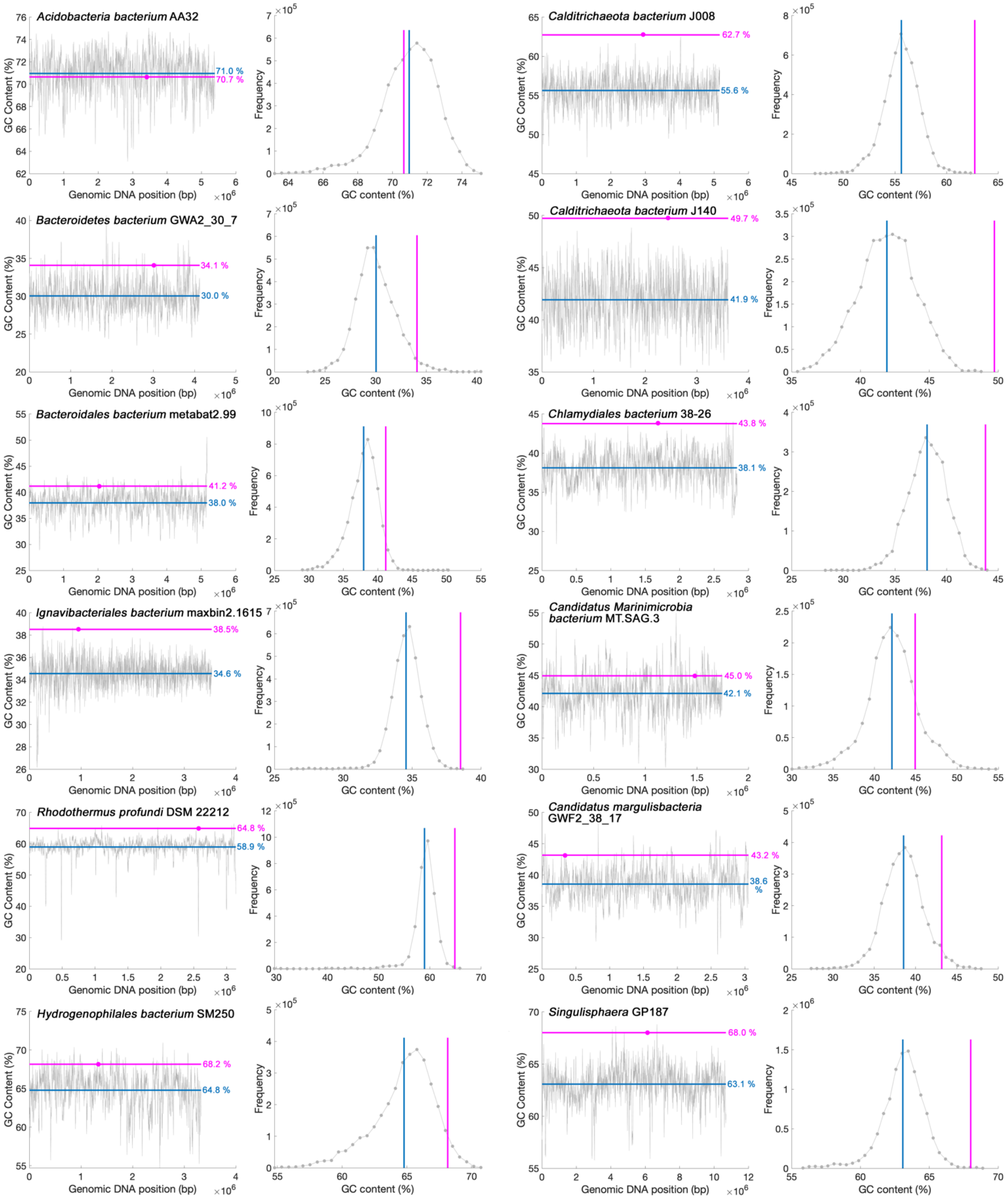

Fig. S10. Genome and CuZnSOD coding sequence GC content analysis for Group 3a CuZnSODs. Left panes show genomic DNA GC content calculated as a moving window average over the whole sequence (grey), total genomic GC content (teal) and GC content of the CuZnSOD coding sequence (pink). Right panes show the GC content distribution for genomic fragments of size approximately equal to that of the CuZnSOD coding sequence (grey) along with whole genome (teal) and CuZnSOD coding sequence (pink) GC content.

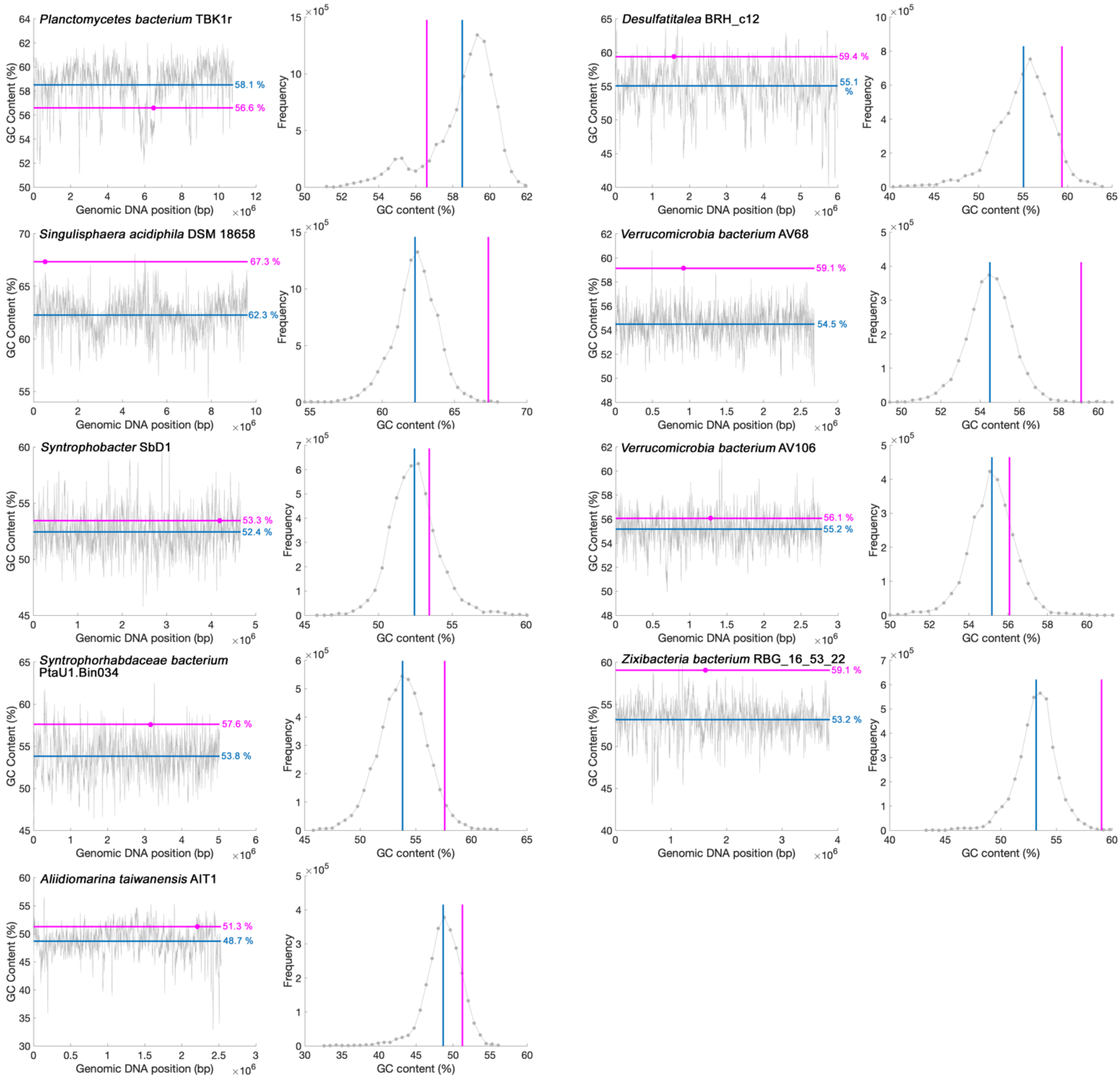

Fig. S10 continued.

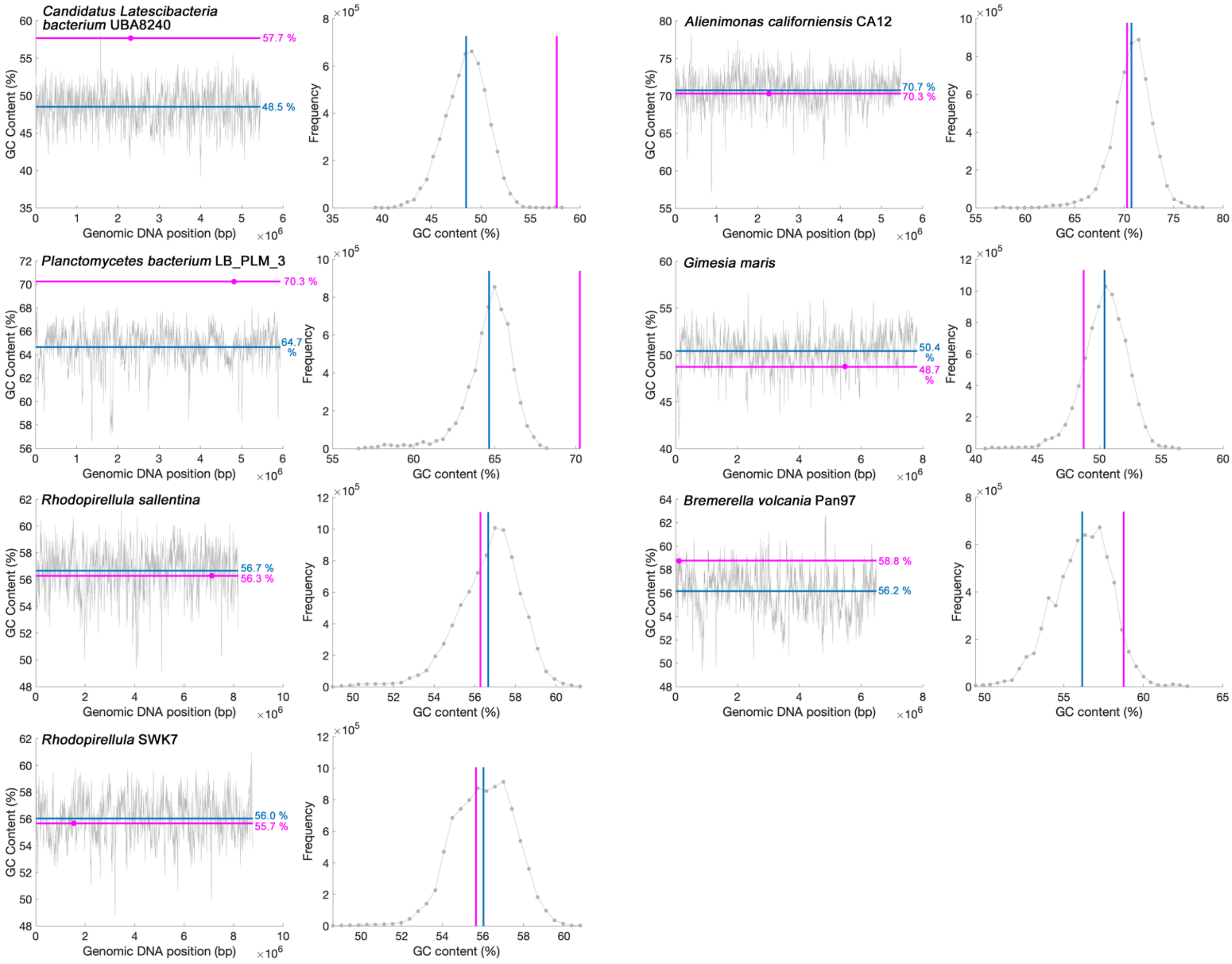

Fig. S11. Genome and CuZnSOD coding sequence GC content analysis for Group 3b CuZnSODs. Left panes show genomic DNA GC content calculated as a moving window average over the whole sequence (grey), total genomic GC content (teal) and GC content of the CuZnSOD coding sequence (pink). Right panes show the GC content distribution for genomic fragments of size approximately equal to that of the CuZnSOD coding sequence (grey) along with whole genome (teal) and CuZnSOD coding sequence (pink) GC content.

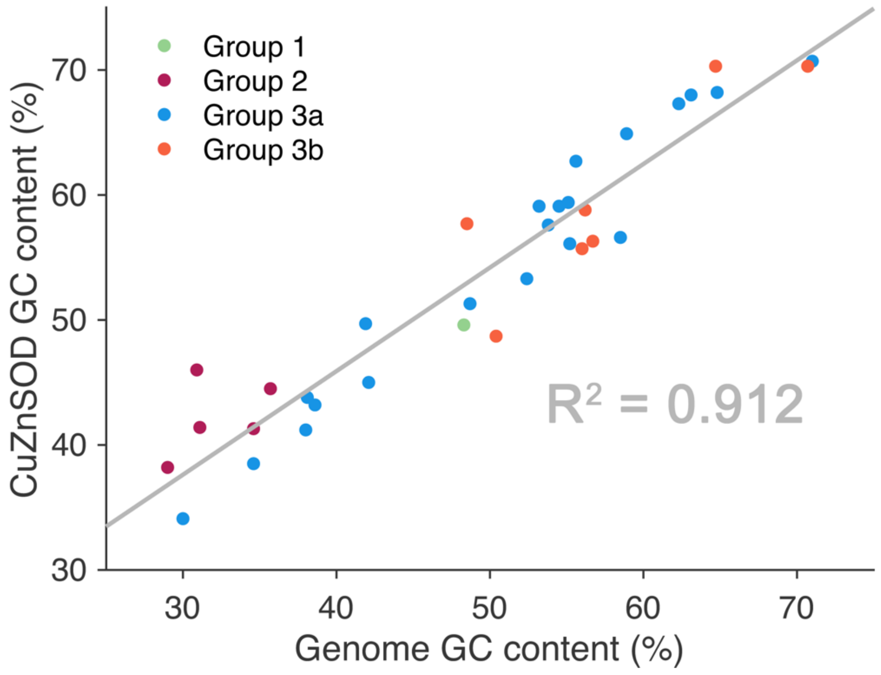

Fig. S12. Relationship between bacterial genome GC content and eukaryotic-like CuZnSOD coding sequence GC content with linear regression. GC content of genomic DNA versus CuZnSOD coding sequences presented in Fig. S8, S9, S10 and S11.

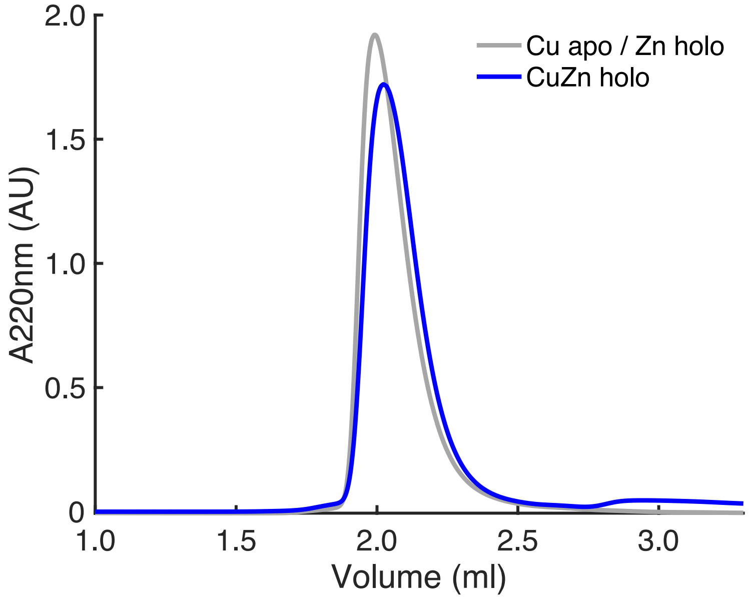

Fig. S13. Addition of copper does not change the quaternary structure of *Bb*CuZnSOD. Size exclusion chromatograms of Cu-apo/Zn-holo (as-isolated) and Cu-holo/Zn-holo *Bb*SOD. Copper metalation slightly reduces the hydrodynamic radius of *Bb*CuZnSOD possibly due to additional constraint of electrostatic and zinc-binding loops.

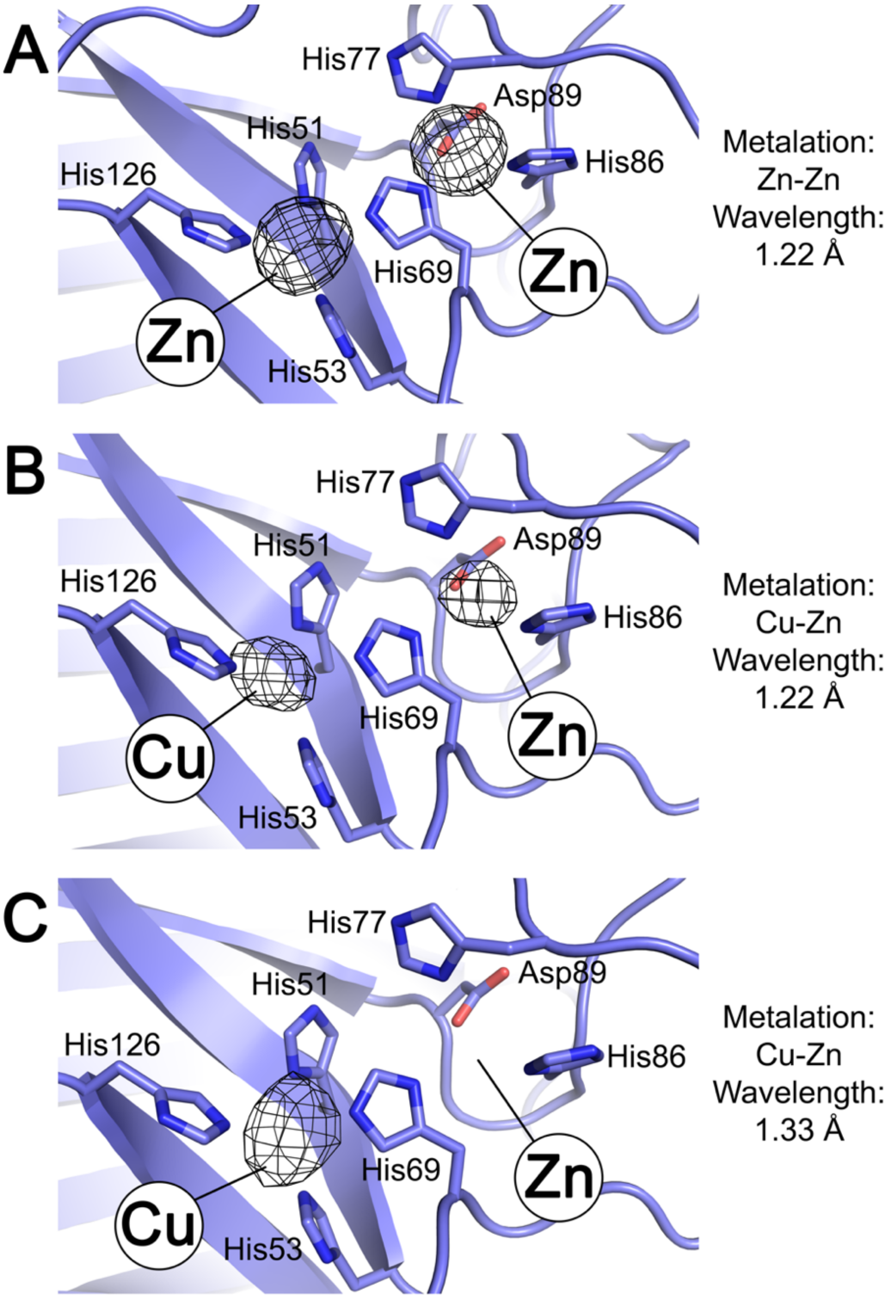

Fig. S14. X-ray characterisation of *Bb*CuZnSOD metal sites. (**A**) As isolated *Bb*CuZnSOD carrying only zinc was crystallised in 2 mM $ZnCl_2$ to yield the Zn-Zn metalated form. Anomalous map calculated from data acquired close to the zinc *K* edge (1.22 Å) indicates the presence of two zinc ions. (**B**) Reconstitution of as-isolated Bb*Zn*SOD with copper yielded dismutase active *Bb*CuZnSOD and was crystallised in the absence of metals. Anomalous map calculated from 1.22 Å data indicated the presence of two metal centres. (**C**) Anomalous map calculated from data collected from the same crystal as (**B**) but close to the copper *K* edge (1.33 Å) shows the presence of only one metal centre indicating the enzyme is correctly Cu-Zn metalated. Anomalous difference maps are calculated from data at 3.0 Å resolution and contoured at 5 σ level.

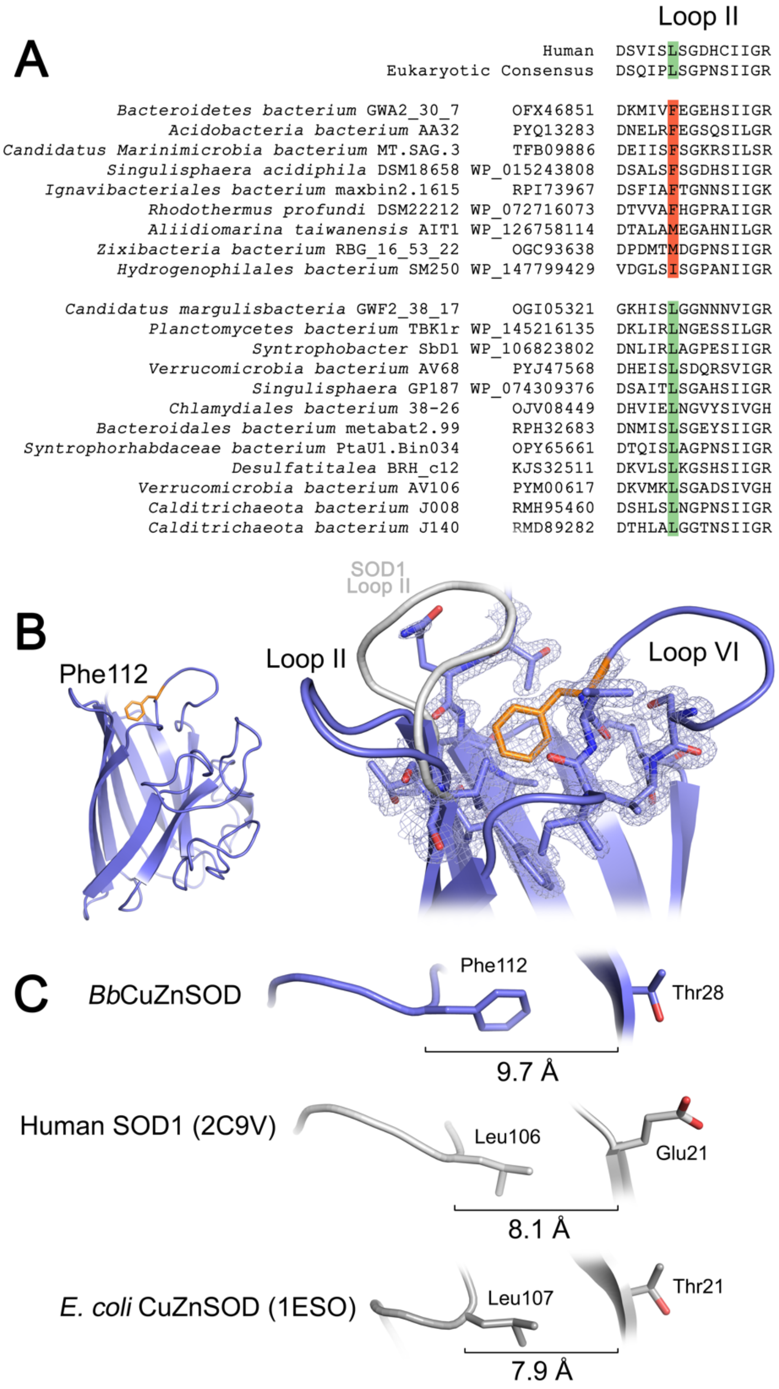

Fig. S15. Destabilisation and deprotection at the *Bb*CuZnSOD β-barrel end. β-barrel plug residues play important roles in CuZnSOD stability. Mutation of human SOD1 Leu38 and Leu106, for example, cause ALS due to disruption of packing in the molecular core. As a result, Leu106 is near ubiquitously conserved across eukaryotic CuZnSODs (98.9%, with only isoleucine and valine as minority variants), bacterial CuZnSODs present in the PDB and Group 1, 2 and 3b E-class prokaryotic CuZnSODs. (**A**) Multiple sequence alignment showing 42% of Group 3a CuZnSODs have destabilising substitutions at the human SOD1 Leu106 site. (**B**) 2Fo-Fc electron density map contoured at 2 σ showing *Bb*CuZnSOD Phe112 at the human SOD1 Leu106 site which introduces 3 additional carbon atoms into the closely packed b-barrel core. *Bb*CuZnSOD Phe112 maintains side-chain orientation and hydrophobic interactions within the β-barrel core but the increased side-chain volume pushes loop VI away from loop II. Furthermore, substitution of human SOD1 Pro28 for *Bb*CuZnSOD Gly33 and removal of two amino acids allows *Bb*CuZnSOD loop II to adopt a tighter structure which does not interact with loop VI in contrast to human SOD1 (grey). Thus, protection of the β-barrel end by loop II is lost. (**C**) Phe112 side-chain repulsion pushes loop VI away from the molecular core as exemplified by an increased Ca-Ca distance between Phe112 and Thr28 in comparison with human and *E. coli* structures.

Supplementary Tables

Table S1. Group 1 *Cyanobacteria bacterium* QH_1_48_107 eukaryotic-like CuZnSOD.

**Organism & Phylum**	**Strain / Isolate**	**CuZnSOD Accession No.**	**Location & date sampled**	**Pubmed ID**	**Monomer size AA / predicted Mr (kDa)**	**Identity to hSOD1**	**Identity to eukaryotic consensus**	**Identity to closest viral or eukaryotic ortholog**	**Identity to *Ph*CuZnSOD**	**Other SODs**	**Signal peptide**
Cyanobacteria bacterium **Cyanobacteria**	QH_1_48_107	PSO53277	Chile: Atacama Desert 2013	28804480	157 / 16.1	58.7%	65.4%	68.0% *Lichtheimia ramosa*	30.8%	Periplasmic P-class: PSO57179.1 Fe-SOD: PSO56206.1	

Table S2. Group 2 eukaryotic-like CuZnSODs.

Organism	Strain / Isolate	CuZnSOD Accession No.	Location	Pubmed iD	Monomer size AA / predicted Mr (kDa)	Identity to hSOD1	Identity to eukaryotic CuZnSOD consensus	Identity to closest viral or eukaryotic ortholog	Identity to *Ph*CuZnSOD	Other SODs	Signal peptide
Candidatus Pelagibacter **Proteobacteria**	TMED142	OUV98905	Mediterranean Sea 2009	28713657	244 / 26.2	54.1%	54.8%	60.7% *Prionace glauca*	33.9%	No P-class CuZn. Fe-SOD: OUV98242	
Pelagibacteraceae bacterium **Proteobacteria**	TMED232	RPG12891	Mediterranean Sea 2009	28713657	190 / 20.2	53.4%	54.1%	60.0% *Prionace glauca*	32.7%	No P-class CuZn, Fe, Mn or Ni SOD.	
Rickettsiales bacterium **Proteobacteria**	TMED131	OUV78940	Mediterranean Sea 2009	28713657	184 / 19.7	56.4%	58.0%	62.5% *Biomphalaria glabrata*	29.8%	No P-class CuZn. Ni-SOD: OUV82477 Fe-SOD: OUV75412	
Rickettsiales bacterium **Proteobacteria**	MED657	MAI84446	South Atlantic Ocean 2010	29337314	221 / 23.6	52.0%	51.7%	58.4% *Ruditapes philippinarum*	32.7%	No P-class CuZn, Fe, Mn or Ni SOD.	
Betaproteobacteria bacterium **Proteobacteria**	TMED41	OUT96107	Mediterranean Sea 2009	28713657	184 / 19.5	51.4%	54.0%	62.3% *Rhizopus delemar* RA 99-880	34.7%	No P-class CuZn, Fe, Mn or Ni SOD.	

Table S3. Group 3a eukaryotic-like CuZnSODs.

Organism	Strain / Isolate	CuZnSOD Accession No.	Location	Pubmed ID	Monomer size AA / predicted Mr (kDa)*	Identity to hSOD1	Identity to eukaryotic CuZnSOD consensus	Identity to closest viral or eukaryotic ortholog	Identity to *Ph*CuZnSOD	Other SODs	Signal peptide
Acidobacteria bacterium **Acidobacteria**	AA32	PYQ13283	USA: Angelo Coast Range Reserve, California (meadow soil) 2014	2989944 4	167 / 16.9	49.0%	51.6%	55.7% Harmonia axyridis	33.6%	No P-class CuZn. Mn/Fe-SOD1: PYQ14901	Sec translocon / Signal Peptidase I
Bacteroidetes bacterium **Bacteroidetes**	GWA2_30_7	OFX46851	USA: Rifle, Colorado 2011	27774985	153 / 16.2	49.4%	49.3%	56.1% *Taenia asiatica*	35.0%	No P-class CuZn. Fe-SOD: OFX58688 Mn-SOD1: OFX42664	Sec translocon / Signal Peptidase I
Bacteroidales bacterium **Bacteroidetes**	metabat2.99	RPH32683	USA: North Dakota, Cottonwood Lake Study Area (Prairie Pothole Region wetland sediments) 2015	30086797	155 / 16.3	47.7%	50.7%	55.3% *Apostichopus japonicus*	34.3%	No P-class CuZn. Fe-SOD: RPH32234	Sec translocon / Signal Peptidase I
Ignavibacteriales bacterium **Bacteroidetes**	maxbin2.1615	RPI73967	USA: North Dakota, Cottonwood Lake Study Area (Prairie Pothole Region wetland sediments) 2015	30086797	173 / 18.5	47.1%	49.7%	51.0% Paelopatides sp. YL-2018	27.4%	No P-class CuZn. Mn-SOD: RPI69506	Sec translocon / Signal Peptidase I
Rhodothermus profundi **Bacteroidetes**	DSM 22212	WP_072716073 (RefSeq)	-	-	168 / 17.1	46.4%	49.0%	58.2% *Agrilus planipennis*	33.3%	No P-class CuZn. Mn-SOD: SHK91031	Lipoprotein signal peptide / Sec translocon/ Signal Peptidase II
Hydrogenophilales bacterium **Proteobacteria**	SM250	TXF12230	Terrestrial volcanic mud	-	162 / 16.0	53.0%	56.3%	56.1% *Tuber aestivum*	32.5%	No P-class CuZn. Fe-SOD: TXF11977	Lipoprotein signal peptide / Sec translocon/ Signal Peptidase II

Calditrichaeota bacterium **Calditrichaeota**	J008	RMH95460	Japan: Shikinejima Island (iron-rich, hot spring) 2016	31413226	162 / 16.8	55.8%	56.4%	60.0% *Calypte anna*	37.7%	No P-class CuZn. Mn-SOD: RMI13018	Lipoprotein signal peptide / Sec translocon/ Signal Peptidase II
Calditrichaeota bacterium **Calditrichaeota**	J140	RMD89282	Japan: Shikinejima Island (iron-rich, hot spring) 2016	31413226	161 / 16.7	49.7%	51.6%	55.2% *Agrilus planipennis*	35.0%	No P-class CuZn. Fe/Mn-SOD: RMD88162	Lipoprotein signal peptide / Sec translocon/ Signal Peptidase II
Chlamydiales bacterium **Chlamydiae**	38-26	OJV08449	South Africa: University of Cape Town (Thiocyanate ramp bioreactor) 2014		156 / 16.0	49.6%	49.0%	56.0% *Equus przewalskii*	37.7%	No P-class CuZn, Fe, Mn or Ni.	Sec translocon / Signal Peptidase I
Candidatus Marinimicrobia bacterium **Marinimicrobia**	MT.SAG.3	TFB09886	Pacific Ocean 2012		161 / 16.3	51.4%	49.4%	55.47% *Colobus angolensis palliatus*	29.3%	No P-class CuZn. Mn-SOD: TFB12340	Lipoprotein signal peptide / Sec translocon/ Signal Peptidase II
Candidatus margulisbacteria **Margulisbacteria**	GWF2_38_17	OGI05321	USA: Rifle, Colorado 2011	27774985	167 / 17.2	49.4%	45.4%	50.7% *Terrapene carolina triunguis*	31.4%	No P-class CuZn, Fe, Mn or Ni.	Lipoprotein signal peptide / Sec translocon/ Signal Peptidase II
Singulisphaera **Planctomycetes**	GP187	WP_074309376 (RefSeq)	-	-	172 / 17.2	53.9%	52.6%	56.0% *Notechis scutatus*	33.6%	No P-class CuZn. Mn-SOD: WP_074305095	Sec translocon / Signal Peptidase I
Planctomycetes bacterium **Planctomycetes**	TBK1r	QDV85848	Pacific Ocean: Lau Basin, Valu Fa ridge, Iron hydroxide deposits 2009	31740763	171 / 18.1	44.52%	49.4%	52.3% *Leptotrom-bidium deliense*	33.3%	No P-class CuZn. Mn-SOD: WP_145219583 Ni-SOD: QDV85089	Possible trans-membrane anchor
Singulisphaera acidiphila **Planctomycetes**	DSM 18658	WP_015243808 (RefSeq)	Russia: sphagnum peat bog	-	172 / 17.4	51.9%	51.3%	53.2% *Notechis scutatus*	32.1%	No P-class CuZn. Mn-SOD: WP_015248067	Sec translocon / Signal Peptidase I
Syntrophobacter **Proteobacteria**	SbD1	WP_106823802 (RefSeq)	-	-	157 / 16.1	46.5%	48.1%	54.9% *Apostichopus japonicus*	34.0%	No P-class CuZn. Fe-SOD: WP_106821012	Lipoprotein signal peptide / Sec translocon/ Signal Peptidase II

Syntrophorhabd-aceae bacterium **Proteobacteria**	PtaU1.Bin034	OPY65661	USA (Lab-scale methanogenic bioreactor treating purified terephthalic acid process wastewater) 2014	28892300	162 / 16.7	50.4%	58.2%	59.9% *Echinostoma caproni*	31.4%	No P-class CuZn. Fe-SOD: OPY67347	Sec translocon / Signal Peptidase I
Aliidiomarina taiwanensis **Proteobacteria**	AIT1	WP_126758114 (RefSeq)	Taiwan: Sea Water	30364313	166 / 17.3	50.0%	47.6%	52.2% *Melipona quadrifasciata*	30.4%	No P-class CuZn. Fe-SOD1: RUO40567	Lipoprotein signal peptide / Sec translocon/ Signal Peptidase II
Desulfatitalea **Proteobacteria**	BRH_c12	KJS32511	Switzerland: Mt Terri URL, St-Ursanne (Opalinus Clay rock porewater BRC-3 borehole 2013	26542073	153 / 15.7	52.1%	52.6%	57.1% *Asbolus verrucosus*	32.9%	P-class CuZn: KJS30673 Mn-SOD: KJS33458	Sec translocon / Signal Peptidase I
Verrucomicrobia bacterium **Verrucomicrobia**	AV68	PYJ47568	USA: Angelo Coast Range Reserve, CA (meadow soil) 2014	29899444	162 / 16.7	51.0%	52.9%	55.6% *Pogona vitticeps*	33.8%	No P-class CuZn. Mn-SOD: PYJ47790	Sec translocon / Signal Peptidase I
Verrucomicrobia bacterium **Verrucomicrobia**	AV106	PYM00617	USA: Angelo Coast Range Reserve, California (meadow soil) 2014	29899444	151 / 15.4	50.3%	54.3%	57.0% *Eisenia andrei*	36.2%	No P-class CuZn. Mn-SOD: PYM02824	Sec translocon / Signal Peptidase I
Zixibacteria bacterium+ **Zixibacteria**	RBG_16_53_2 2	OGC93638	USA: Rifle, Colorado 2007	27774985	170 / 17.4	47.7%	53.6%	55.3% *Rhizopus stolonifer*	33.6%	No P-class CuZn. Fe-SOD: OGC92723	No identifiable N-terminal localisation peptide

* Sizes are stated after cleavage of N-terminal signal peptides.

+ The *Zixibacteria bacterium* RBG_16_53_22 (OGC93638) CuZnSOD has conserved E-class dimer interface and an extended N-terminus but no recognisable localisation signal. The similarities between this protein and other group 3a members are clear but it may be that this recently discovered group of bacteria recognise atypical transport signals.

Table S4. Group 3b eukaryotic-like CuZnSODs.

Organism	Strain / Isolate	CuZnSOD Accession No.	Location	Pubmed ID	Monomer size AA / predicted Mr (kDa)	Identity to hSOD1	Identity to eukaryotic CuZnSOD consensus	Identity to closest viral or eukaryotic ortholog	Identity to *Ph*CuZnSOD	Other SODs	Signal peptide
Candidatus Latescibacteria bacterium **Latescibacteria**	UBA8240	HCQ01722	Hydrothermal vent	30148503	173 / 17.5	47.5%	51.3%	54.7% *Loxodonta africana*	34.3%	No P-class CuZn. Mn-SOD: HCQ00464	
Planctomycetes bacterium **Planctomycetes**	LB_PLM_3	REK09122	Saudi Arabia: Thuwal, Red Sea RO plant (biofilm) 2016		185 / 19.0	49.0%	55.2%	58.4% *Sclerodermus guani*	34.3%	No P-class CuZn. Mn-SOD: REK15936	
Rhodopirellula sallentina **Planctomycetes**	-	WP_008687248 (RefSeq)	Italy: Adriatic Sea 2006	23273849	193 / 20.3	47.37%	53.1%	58.8% *Apostichopus japonicus*	34.1%	No P-class CuZn. Ni-SOD: WP_008682318 MnSOD: WP_173403166	Lipoprotein signal peptide
Rhodopirellula **Planctomycetes**	SWK7	WP_009094724 (RefSeq)	Sweden: The Skagerrak 2006	23273849	187 / 19.6	49.0%	54.5%	59.0% *Apostichopus japonicus*	35.5%	No P-class CuZn. Mn-SOD: EMI45100	Lipoprotein signal peptide
Alienimonas californiensis **Planctomycetes**	CA12	QDT15684	USA: Monterey Bay (marine) 2014		150 / 16.5	55.7%	58.6%	58.1% *Macrostomum lignano*	37.8%	No P-class CuZn. Mn-SOD: QDT16147	TAT signal peptide
Gimesia maris **Planctomycetes**	-	WP_002649112 (RefSeq)	Northwest Indian Ocean 2013	-	187 / 19.9	50.7%	54.3%	54.0% *Argopecten irradians*	33.3%	No P-class CuZnSOD. Mn-SOD: WP_002648522	Lipoprotein signal peptide
Bremerella volcania **Planctomycetes**	Pan97	QDU73047	Italy: Panarea Volcanic red biofilm 2013	31894496	172 / 17.7	44.6%	50.0%	51.6% *Aedes albopictus*	36.6%	No P-class CuZnSOD Mn-SOD: WP_144971235	Sec translocon / Signal Peptidase I

Table S5. Bacterial E-class CuZnSOD sequences compiled by phylum.

Phylum	Number of eukaryotic CuZnSODs
Proteobacteria	10
Planctomycetes	9
Bacteroidetes	4
Calditrichaeota	2
Verrucomicrobia	2
Acidobacteria	1
Chlamydiae	1
Cyanobacteria	1
Latescibacteria	1
Margulisbacteria	1
Marinimicrobia	1
Zixibacteria	1

Table S6. Summary of bacterial eukaryotic CuZnSODs characteristics.

	P-Class interface	E-class interface	Disulphide configuration	N-terminal extension
**Group 1**	No	Yes	Eukaryotic	None
**Group 2**	No	No	Eukaryotic	CxRTxAxxCxC motif
**Group 3a**	No	Yes	Prokaryotic	Periplasmic signal peptide
**Group 3b**	No	3/7	No disulphide	Variable

Table S7. Analysis of protein coding sequences immediately adjacent to the *Cb*CuZnSOD coding sequence.

**Name: *Cb*CuZnSOD**	**Accession: PSO53277**	**Contig: PXPJ01000150**
**3’ Protein Coding Sequence**		
ATGCGTAGGCTCTTAACTGTGGGCAAACCAGGTGAACCCCAGTTGCCTACTCCCGAAGTTCTGCGTAATGTCCTAGTGGAGTTGGGTCCGGTCTACATTAAACTAGGACAATTGCTGAGTACTCGCCCTGACTTACTTCCACCAGAATATATCGAAGC<br>CCTCAGCGCCCTCCAAGGGAACGTTCCATCCGTACCGTGGTCTGAAGTGGAAGTTTTAATCCGACGGCAGGTACAAAAGCCTCTCGAAGAGGTGTTTACCCATATCGAGCAAGAACCAGTTGCTGCCGCTTCCATTGCCCAAACTCACAAGGCAATCT<br>TAAGTGATGGTCAAGAAGTGGCGATGAAAGTCCAGCGCCCAGGAATCGACAAGATTGTCGAGCAAGATATCTCTCTGATTAAGGGACTAGCCGAGTTAGTTGCTCGCACTGATTTTGGACAAAATTACGATGTTGTTGCTTTAGCTGAGGAGTTCACC<br>AACGCGCTTCGAGCAGAGCTAGACTTTACCAAAGAAGCTAGCAACACCGACCAATTGCGGCGTAATCTCTCAGCAAGCCGCTGGTTTGACTCGAAGCAGCTCGTCGTGCCCGCAATTTACTGGGATTTGACCACAGAAAAGTTACTGGTGATGGAGTG<br>GCTCCCGGGAAAACCCATTCTCGAAGCGGATATCTCGGCATCGCAAAATGGAGCTGAAAGCAACCAAGGTCGGCAAGAAATTACCACCCTTTTGTTTCGCGCCTTTTTTCAGCAGATTTATATCGACGGCTTCTTCCATGCTGACCCCCATCCCGGCA<br>ACTTATTTTATCTCCAAGACGGTCGGGTGGACTGCTCGATTGTGGCATGATTGGACGTCTTGACCCACGCACGCAGCAGATTTTGACCGAGATGCTGTTGGCAACTGTCGAGATTGATGCCCAGCGGTGTAGTGAATTGACGTTGGAGCTGGCTGAG<br>TCGGGACAGCCTGTGAGTTTAGCGCGTTTGGAAAACGATTACGACCGGATGCTGCGCAAATATCACAACCTAAGCATATCAGAAATTAACTTCAGCCAGGTGTTTTATGAGATCTTGCAAGTCTCGCGCGACAATAAAGTCAAGTTACCCAGCAATAT<br>GGGGTTATATGCCAAGAGCCTAGCCAACCTGGAGGGAGTTGCCCGTGGTTTCAACCCAGAGGTGAATCTATTAGAGGAGATTAAACCATTGATGAGTGACCTATTACGGCGCCAGTTGCTTGGGGAGAGTCCACTCAACACCTTTCTGAGAACCGCTC<br>TTGACCTCAAAACTCTTTCTTTGCAGTCTCCGAGGCAACTTGAGCAGCTTTTAGACCGCATCACCTCCGAAACTCTGACTTGGAACTTGACCCTACAAGAACTGGGTCCGCTACGTCGCAGTGTGGATGATTCAGCGAATCGACTTTCGTTTAGCGTG<br>GTAGTAGGTTCGTTGATTATCGGCGCGGCAATTATTTCTTCTAACGCTCAAACTACCCAGCTATCTTGGATTAGCACTTTCCTCTTCGCGGCTGCCAGCTTCATCGGACTGTGGCTGATTATCAGTATTTGGCGCTCTGGGCGTTTGAAGTAA		
**Translated Protein Sequence**	**Description: AarF/ABC1/UbiB kinase family protein**	
MRRLLTVGKPGEPQLPTPEVLRNVLVELGPVYIKLGQLLSTRPDLLPPEYIEALSALQGNVPSVPWSEVEVLIRRQVQKPLEEVFTHIEQEPVAAASIAQTHKAILSDGQEVAMKVQRPGIDKIVEQDISLIKGLAELVARTDFGQNYDVVALAEEFT<br>NALRAELDFTKEASNTDQLRRNLSASRWFDSKQLVVPAIYWDLTTEKLLVMEWLPGKPILEADISASQNGAESNQGRQEITTLLFRAFFQQIYIDGFFHADPHPGNLFYLQDGRVGLLDCGMIGRLDPRTQQILTEMLLATVEIDAQRCSELTLELAE<br>SGQPVSLARLENDYDRMLRKYHNLSISEINFSQVFYEILQVSRDNKVKLPSNMGLYAKSLANLEGVARGFNPEVNLLEEIKPLMSDLLRRQLLGESPLNTFLRTALDLKTLSLQSPRQLEQLLDRITSETLTWNLTLQELGPLRRSVDDSANRLSFSV<br>VVGSLIIGAAIISSNAQTTQLSWISTFLFAAASFIGLWLIISIWRSGRLK		
**Closest Bacterial Orthologue**		
WP_006101422	Blast Score 835	Identity **76%**
Coleofasciculus chthonoplastes	Cyanobacteria	
**Closest Non-Bacterial Orthologue**		
N/A		

**5’ Protein Coding Sequence**		
ATGAGAAAAACCATTATCGGTGTGATGGGGCCAGGAGATGGGGCTTCAGTAGCAGACAGAGAAAATGCCTACGAACTCGGTAAACTCATTGCTACTCAGGGATGGGTGTTGCTGACGGGTGGCAGGAAGGAAGGAGTAATGGATGCGGCTAACAAAGG<br>CGCAAAAGCTGCCGATGGCTTAACCCTGGGAATATTACCGACTAATGATTCTCATGCTGTCTCAGGATCGGTAGACTTGGCAGTTTTTACAGAGATGGGTAATGCTCGCAACAATATCAATGTTCTCTCTAGCGACGTAGTGATTGCCTGTGGCATGG<br>GGATGGGAACCGCTTCGGAAGTTGGTCTTGCCTTGAAAGCAGGCAAAAAGGTGGTTTTGTTAACTAACCATCAACCCACTCAGCAGTTTTTTAGCGGTTTTTCACCAGATAATGTTTTTGTGGCAACTTCTCCAGAAACTGCACTCACCTTAGTTAAG<br>GAAACTTTAACCTATAATTTTTAA		
**Translated Protein Sequence**	**Description: TIGR00725 family protein**	
MRKTIIGVMGPGDGASVADRENAYELGKLIATQGWVLLTGGRKEGVMDAANKGAKAADGLTLGILPTNDSHAVSGSVDLAVFTEMGNARNNINVLSSDVVIACGMGMGTASEVGLALKAGKKVVLLTNHQPTQQFFSGFSPDNVFVATSPETALTLVK<br>ETLTYNF		
**Closest Bacterial Orthologue**		
WP_075903022.1	Blast Score 221	Identity **65%**
Moorea bouillonii	Cyanobacteria	
**Closest Non-Bacterial Orthologue**		
WP_048092690.1	Blast Score 93	Identity **39%**
Geoglobus acetivorans	Archaea	

Table S8. Analysis of protein coding sequences immediately adjacent to the *Rb*CuZnSOD coding sequence.

**Name: *Rb*CuZnSOD**	**Accession: OUV78940**	**Contig: NHGG01000066**
**3' Protein Coding Sequence**		
ATGACAGCAATAGTGCAACTAACAGAAACAGCAAAGGAGCATATGGCTACAATGCTAAAAGAACACGATAAGCCGGCTATTAGGTTAGGATTAAAAGGCGGTGGCTGTGCGGGTTTCAAATACGAATGGAGTCTTGAAGACGAAATCAGGAGCGATGA<br>CGAACAAATTAAGGTAGATGGAGGTTTGTTTGTTGTAGATCCTGCGAGTGTTATGTACTTATTAGGTACTACGATAGATTATAAGAAAGAAGTATTTGGATCATATTTTGATATTAAAAGCCCTAATGCAACATCAAGTTGCGGTTGTGGCGAATCAG<br>TAGGATTTTAA		
**Translated Protein Sequence**	**Description: Iron-sulfur cluster insertion protein ErpA**	
MTAIVQLTETAKEHMATMLKEHDKPAIRLGLKGGGCAGFKYEWSLEDEIRSDDEQIKVDGGLFVVDPASVMYLLGTTIDYKKEVFGSYFDIKSPNATSSCGCGESVGF		
**Closest Bacterial Orthologue**		
WP_009801260.1	Blast Score 100	Identity **43%**
Oceanicaulis sp. HTCC2633	Proteobacteria	
**Closest Non-Bacterial Orthologue**		
XP_021677118.1	Blast Score 91	Identity **40%**
Hevea brasiliensis	Eukaryote	

**5' Protein Coding Sequence**		
atggttaggttgggaagtgacgtcctacctgtttatactgagcctacttggtcttacttctggcgcaagcctgatgtacgaaaatattgcgagaccaacttcgctcacgtcggcgcaaaagctcgtgaacttgatgttaggttgtctatgcatcctgg<br>tcagtttactgtacttgcgtcagataacccagatatagtaaatagaagcatagaggagtttgagtatcatgttgattgcatcagatggatgggctacggccaacaattccaagactttaaatgtaatatccacatatcaggcaggcaaggtccacccg<br>gtatcatcaatgctctcccaagattatctcaagaggcgagaaacgttattacgatcgagaacgacgaaatgtcgtggggcatcgatgcgtcactcgaacttgagaaacatgtcgcactcgtacttgacatacaccatcactgggtgcgtacaggtgaa<br>tacatacaaccctccgacgatagatatcatcgcgtagttgacagttggcgtggtgtacgtcctgttatccattacagtgtttcacgtgaagacttacttgtaggtcacgatgctgatacacttcctaacatggacgaactacttgaacaaggctttaa<br>gaaacaaaaactacgagctcatagcgatatgatgtggaataacgcagtaaatgactgggctctactgtttaacgataccgcagacattatggtagagtctaaacacaagaaccttgcttcgcagaaactgttagaacataaggtaaatacagtatgcg<br>atttaaacaacttcaaaactgtgaacgtaccaaagcaagaacttgtcagtgcgaaagcctaa		
**Translated Protein Sequence**		
MVRLGSDVLPVYTEPTWSYFWRKPDVRKYCETNFAHVGAKARELDVRLSMHPGQFTVLASDNPDIVNRSIEEFEYHVDCIRWMGYGQQFQDFKCNIHISGRQGPPGIINALPRLSQEARNVITIENDEMSWGIDASLELEKHVALVLDIHHHWVRTGE<br>YIQPSDDRYHRVVDSWRGVRPVIHYSVSREDLLVGHDADTLPNMDELLEQGFKKQKLRAHSDMMWNNAVNDWALLFNDTADIMVESKHKNLASQKLLEHKVNTVCDLNNFKTVNVPKQELVSAKA		
**Closest Bacterial Orthologue**	**Predicted function: UV damage endonuclease UvsE**	
WP_160116215.1	Blast Score 355	Identity **67%**
Legionella busanensis	Proteobacteria	
**Closest Non-Bacterial Orthologue**		
YP_009811715.1	Blast Score 259	Identity **51%**
Dickeya phage vB_DsoM_AD1	Virus	

Table S9. Analysis of protein coding sequences immediately adjacent to the *Bb*CuZnSOD coding sequence.

**Name: *Bb*CuZnSOD**	**Accession: OFX46851**	**Contig: MENC01000241**
**3' Protein Coding Sequence**		
atgaataaaatcttacttataattttaataatctgtttgtttacaaaatcttatgctcaaaataacgaaattgattcgttattaaaagtaatcaaattcacaaaaaacgattcccttaaagtaaaagatttattatctctttcaagtttatataccga<br>caaaaaccttgattctgcaataatatttgcaaaacaggctatcgaaatttcaaaaaaaaataacaataaaaaacaattgtcaactgcttatgtagaacttggatgggcaatttacatgatgggaaactattctgaggcattgattaattactttgaag<br>cacttaaaattagtgaatcaattaatgacaaaaagcagatttcaaattcacttggaaatattggtgcagtttataaaactatgaatgaccttcaaaaagccaaagattattactttaaagctttgaaaatagacgaagaattagctgtaaaagaatgg<br>attgctattgatataagtaatttgggaataatttatatggaagaaggagattgttcaaaagctctggaattttatttcaaagcattgaaaataaacgaagagatgaataataaacaagaaatttcaattaagcttggaaatattgcaaatgtttatct<br>gaaacttggcgaagcgattgaaaatcagccaaaaactcaagacagtctttataatcttgctctggaatattattttaaagctcttaaaattgatgaagaaattggtcgtaaaagcggaatcgttgttaaatatgcaaatattggcaacttatacatga<br>atattcaaaaatatttaaaagccaaagaatatcttttaaaagcccttgagctttcatatagtattggtatattggattataccgaaagcatacataaaagtctaagccagatttatgattcattgaaaatacctcaaaaagcgttagaacattacaaa<br>ctatatgttttatacaaagacagcatttttaacgaagaagcaaccaaaaattctgttcgcattgaaatgaattttgagtttgaaaaaaaacaagcagtagaaaaagccgaacaggaaaaaaaagatgcaattcaaaaagcagaagaaaacaaacaacg<br>attaatcctaatatttgtgtcacttgggcttatgcttgttgcagcattttcaattttatattcaaaatgtttattcagaaaaaaaaggcaaatatattattgagcaatcaaaatattgaaataaaacagcaaaaggaagaaatacagtctcaacgag<br>atgaaatagaggcacaaagagatatggtaacatttcaaaaagaagaaattgaaggaagtataaaatatgcacaaataattcaaaatgcagttttgccttctcagcagcttgctaaagatttgttcggcgaatatttcatattgttcaaaccaaaagat<br>gttgtaagtggagatttttactggttttcaaaagtaaacaactggactttaattgcagttgcagattgtaccggacatggagttccgggcggatttatgagtatgttagggataagcttttaaacgaaattgttgcaagaaatgaagttcaaacagc<br>atcttctgtacttgaagaaatgcgaaaatatgtaatccagtcgcttcagcagcaatctacaaatgaagataattttaaaattgattacaattcagaaaatctgaacattaaagacggaatggatatgagtttcgttgcaattgattttaatacaggga<br>aaattcaatttgcgggagctaataattcgctttggatagtaaaaattgaaagtgagaaagtgagactcggtgaaagtgtgactgaaaatcctctcactttctcactttcgcaatctcccactttctcttcattaaatgaaaccaatttactgattgaa<br>gtaaagcccgacaagcaaccagttgcaatttacgaaagcatgaaacctttcacaaaccatgaaatacaactccaaaaaggtgatataatttatctttttcagatggttattccgaccagtttggcggacctaaaggcaaaaaatttaaaagtcgtgt<br>gtttcaaaatattataaaagaaaatgcacaattacctatgaacgagcaatgtgtcaaactaaactcagttattgaaaattggaaaaacgcacattccgaaaaacatcatcaaactgacgatattacagttttaggtataaaatatttataa		
**Translated Protein Sequence**	**Description: Tetratricopeptide repeat protein**	
MNKILLIILIICLFTKSYAQNNEIDSLLKVIKFTKNDSLKVKDLLSLSSLYTDKNLDSAIIFAKQAIEISKKNNNKKQLSTAYVELGWAIYMMGNYSEALINYFEALKISESINDKKQISNSLGNIGAVYKTMNDLQKAKDYYFKALKIDEELAVKEW<br>IAIDISNLGIIYMEEGDCSKALEFYFKALKINEEMNNKQEISIKLGNIANVYLKLGEAIENQPKTQDSLYNLALEYYFKALKIDEEIGRKSGIVVKYANIGNLYMNIQKYLKAKEYLLKALELSYSIGILDYTESIHKSLSQIYDSLKIPQKALEHYK<br>LYVLYKDSIFNEEATKNSVRIEMNFEFEKKQAVEKAEQEKKDAIQKAEENKQRLILIFVSLGLMLVAAFSIFIFKMFIQKKKANILLSNQNIEIKQQKEEIQSQRDEIEAQRDMVTFQKEEIEGSIKYAQIIQNAVLPSQQLAKDLFGEYFILFKPKD<br>VVSGDFYWFSKVNNWTLIAVADCTGHGVPGGFMSMLGISFLNEIVARNEVQTASSVLEEMRKYVIQSLQQQSTNEDNFKIDYNSENLNIKDGMDMSFVAIDFNTGKIQFAGANNSLWIVKIESEKVRLGESVTENPLTFSLSQSPTFSSLNETNLLIE<br>VKPDKQPVAIYESMKPFTNHEIQLQKGDIIYLFSDGYSDQFGGPKGKKFKSRVFQNIIKENAQLPMNEQCVKLNSVIENWKNAHSEKHHQTDDITVLGIKYL		
**Closest Bacterial Orthologue**		
WP_092438716.1	Blast Score 449	Identity **38%**
Williamwhitmania taraxaci	Bacteroidetes	
**Closest Non-Bacterial Orthologue**		
WP_052718337.1	Blast Score 147	Identity **35%**
Methanosarcina sp. MTP4	Archaea	

**5' Protein Coding Sequence**		
atgaaactaattcaagacataattaacgaactcattgatattgataagtcaattagttcgccgtttttaaaaaccaaagttcttgcaagcagactacaaaaccaagtgcttttaaattgggtaagtaatgaacttaaaggttacgacaattcgaatat<br>tatacccaattatagaaaatataaaggaaatattacaggaacttatataaatgggagttaccaatttaatgaccaaccagttccgacaaatggacttgaacctgagtttgtggaagaattacattcaatgaacttttatcaaagcatagtaagtcttg<br>aaacacttaaaagagaaaataaatctggaaaattggaacatacattttcagctgaacgaattggactaattcagcaaaattggaaaaaaatgggaaatccgtatttgcttttgattaattgtaaaatatccattccagttaatactgttgttgaaata<br>ctctcttatgttagaaataacttacttgactttatgcttaaaattgattccgaatttgggaacattacagaaatagaagaactaaaaactaaaaaagaagaaattgcaactattatgaatcagactattataaataattcaggagacggaaatgttgt<br>aaataccggagaaaatgcgaaaatatcagcaactataagtatcacaaaaggaagtaaagaagaattagtaaaacatttgctggacaaaggtctgagcaaagttgacacagcagaacttgttgagataattgataccgaagagcctaactttggaaata<br>aaacattcggacaaaaagtaaactcn		
**Translated Protein Sequence**	**Description: N/A**	
MKLIQDIINELIDIDKSISSPFLKTKVLASRLQNQVLLNWVSNELKGYDNSNIIPNYRKYKGNITGTYINGSYQFNDQPVPTNGLEPEFVEELHSMNFYQSIVSLETLKRENKSGKLEHTFSAERIGLIQQNWKKMGNPYLLLINCKISIPVNTVVEI<br>LSYVRNNLLDFMLKIDSEFGNITEIEELKTKKEEIATIMNQTIINNSGDGNVVNTGENAKISATISITKGSKEELVKHLLDKGLSKVDTAELVEIIDTEEPNFGNKTFGQKVN		
**Closest Bacterial Orthologue**		
WP_062300492.1	Blast Score 435	Identity **82%**

Roseivirga seohaensis	Bacteroidetes	
**Closest Non-Bacterial Orthologue**		
WP_048141644.1	Blast Score 79	Identity **29%**
Methanosarcina horonobensis	Archaea	

Table S10. Crystallographic data collection and refinement statistics

**Metalation**	**Zn-Zn[#]**	**Cu-Zn[#]**
**PDB**	7B4O	7B4P
**Data collection**		
Space group	$C222_1$	$P2_12_12_1$
**Cell dimensions**		
a, *b*, *c* (Å)	96.5, 96.6, 136.1	41.8, 109.6, 122.6
α, β, γ (°)	90, 90, 90	90, 90, 90
Resolution (Å)*	68.3 – 1.41 (1.43 – 1.41)	81.7 - 2.7 (2.83 - 2.7)
No. reflections	122059	16194
R_{merge}*	10.7 (207)	10.6 (231)
$I / \sigma I$*	10.2 (0.9)	10.6 (0.8)
CC1/2	0.998 (0.345)	0.999 (0.606)
Completeness (%)*	100 (100)	100 (99.9)
Redundancy*	7.5 (7.6)	9.2 (9.0)
Wilson B-factor (Å^{-2})	11.8	87.5
**Refinement**		
ASU content	2 dimers	2 dimers
Resolution (Å)	68.37 – 1.41	81.8-2.7
No. reflections	115779	15343
R_{work} / R_{free} (%)	16.0 / 19.2	24.0 / 27.7
**No. of atoms**		
Protein	4559	4422
Water	564	21
No. of residues	592	589
**Average *B*-factors (Å^2)**		
Protein	17.5	97.2
Water	32.6	71.6
**R.M.S. deviations**		
Bond lengths (Å)	0.0074	0.0021
Bond angles (°)	1.36	1.22

[#] Each data set is taken from a single crystal.

* Values in parenthesis refer to the highest resolution shell.

Table S11. Archaeal CuZnSODs. All currently known examples of archaeal CuZnSODs are restricted to two methanogen genera Methanocellales and Methanosarcinales. Each enzyme exhibits P-class intra-subunit disulphide configuration indicating the presence of an ancestral bacterial CuZnSOD during the early stages or archaeal evolution, or, lateral gene transfer from bacteria as Methanomicrobia diversified. Given the lack of CuZnSODs in other archaeal classes, the latter appears more likely.

Class	Genus	Species & strain	Genbank Accession
Methanomicrobia	Methanocellales	*Methanocella arvoryzae* MRE50	CAJ35168
Methanomicrobia	Methanosarcinales	*Methanosarcina acetivorans* C2A	AAM05808
Methanomicrobia	Methanosarcinales	*Methanosarcina barkeri*	WP_011307313
Methanomicrobia	Methanosarcinales	*Methanosarcina barkeri* MS	AKB54462
Methanomicrobia	Methanosarcinales	*Methanosarcina thermophila*	WP_048166087
Methanomicrobia	Methanosarcinales	*Methanosarcina flavescens*	WP_054297776
Methanomicrobia	Methanosarcinales	*Methanosarcina* sp. MSH10X1	WP_128504894
Methanomicrobia	Methanosarcinales	*Methanosarcina siciliae*	WP_052721759
Methanomicrobia	Methanosarcinales	*Methanosarcina siciliae* T4/M	AKB28179
Methanomicrobia	Methanosarcinales	*Methanosarcina siciliae* C2J	AKB36155
Methanomicrobia	Methanosarcinales	*Methanosarcina*	WP_048122945
Methanomicrobia	Methanosarcinales	*Methanosarcina* sp. Ant1	OEU42080
Methanomicrobia	Methanosarcinales	*Methanosarcina* sp. WWM596	AKB18164
Methanomicrobia	Methanosarcinales	*Methanosarcina spelaei*	WP_095643034
Methanomicrobia	Methanosarcinales	*Methanosarcina horonobensis*	WP_048139791
